# Acceptability of risk stratification within population‐based cancer screening from the perspective of the general public: A mixed‐methods systematic review

**DOI:** 10.1111/hex.13739

**Published:** 2023-02-28

**Authors:** Lily C. Taylor, Alison Hutchinson, Katie Law, Veeraj Shah, Juliet A. Usher‐Smith, Rebecca A. Dennison

**Affiliations:** ^1^ The Primary Care Unit, Department of Public Health and Primary Care, School of Clinical Medicine University of Cambridge Cambridge UK; ^2^ School of Clinical Medicine University of Cambridge Cambridge UK

**Keywords:** acceptability, cancer, mixed methods, risk stratification, screening, systematic review

## Abstract

**Introduction:**

Risk‐stratified cancer screening has the potential to improve resource allocation and the balance of harms and benefits by targeting those most likely to benefit. Public acceptability has implications for engagement, uptake and the success of such a programme. Therefore, this review seeks to understand whether risk stratification of population‐based cancer screening programmes is acceptable to the general public and in what context.

**Methods:**

Four electronic databases were searched from January 2010 to November 2021. Qualitative, quantitative and mixed‐methods papers were eligible for inclusion. The Joanna Briggs Institute convergent integrated approach was used to synthesize the findings and the quality of included literature was assessed using the Mixed Methods Appraisal Tool. The Theoretical Framework of Acceptability was used as a coding frame for thematic analysis. PROSPERO record 2021 CRD42021286667.

**Results:**

The search returned 12,039 citations, 22 of which were eligible for inclusion. The majority of studies related to breast cancer screening; other cancer types included ovarian, kidney, colorectal and prostate cancer. Risk stratification was generally acceptable to the public, who considered it to be logical and of wider benefit than existing screening practices. We identified 10 priorities for implementation across four key areas: addressing public information needs; understanding communication preferences for risk estimates; mitigating barriers to accessibility to avoid exacerbating inequalities; and the role of healthcare professionals in relation to supporting reduced screening for low‐risk individuals.

**Conclusion:**

The public generally find risk stratification of population‐based cancer screening programmes to be acceptable; however, we have identified areas that would improve implementation and require further consideration.

**Patient or Public Contribution:**

This paper is a systematic review and did not formally involve patients or the public; however, three patient and public involvement members were consulted on the topic and scope before the review commenced.

## 1 INTRODUCTION

Cancer screening programmes have been implemented in many high‐income countries aiming to reduce incidence and/or mortality through prevention, early detection and treatment.^1^ Most programmes operate a fixed regime, with eligibility determined by age and/or sex and screening intervals determined by the screening test result.^2^ However, the population are not all at equal risk of developing cancer as cancer risk is associated with individual‐level characteristics, including lifestyle and genetic factors.^3^ Risk stratification represents an opportunity to improve the balance of harms and benefits by targeting those most likely to benefit, whilst optimizing distribution of limited healthcare resources.^2^, ^4^, ^5^ For example, low‐risk individuals could be invited less frequently, at a later age or forgo screening completely. Conversely, high‐risk groups could be invited at an earlier age, more often or be referred for colonoscopy at lower thresholds.^4^

With risk stratification, the advantages of earlier detection and treatment are maximized for those at high‐risk, whereas screening‐related harms such as false‐positive or false‐negative test results, physical or psychological harm, overdiagnosis and overtreatment are reduced among lower‐risk cohorts.^1^, ^6^ Additionally, personal risk information may confer benefits for screening decision‐making and opportunities to engage in primary prevention and risk management, such as lifestyle change, risk‐reducing medication or prophylactic surgery.^2^ A multitude of risk prediction models have been developed across different cancer types using a variety of risk factors.^7^, ^8^, ^9^, ^10^

Risk stratification incurs ethical challenges associated with introducing systematic differences between population subgroups, including concerns about equity and accessibility, for example, which risk factors to include in risk modelling, including modifiable factors like alcohol consumption or smoking, and how to screen participants with missing data. Public acceptability is fundamental to success, as it has direct implications for uptake and adherence.^11^ There is a need to elucidate areas of misunderstanding and optimal communication strategies ahead of implementation to ensure acceptability at a population level. Unlike previous reviews that have focussed on breast cancer,^12^, ^13^ this review takes a broader perspective and aims to explore whether risk stratification within population‐based screening programmes for any cancer is acceptable to the general public and in what context.

## 2 METHODS

This literature review was performed in tandem with one considering the perspectives of healthcare professionals (HCPs),^14^ with one literature search and title and abstract screening performed for both reviews (PROSPERO 2021 CRD42021286667).

### 2.1 Search strategy

We searched MEDLINE, Embase, Web of Science and PsycINFO electronic databases using title and abstract search terms and MeSH terms (the full search strategy is presented in Supporting Information: Table S1). We restricted the search date from 1 January 2010 to 31 November 2021 in response to preliminary searches and to ensure contemporary views of the public in relation to risk‐stratified screening, considering advances in genomics and risk prediction modelling over the past decade.^15^, ^16^

### 2.2 Study selection

We defined risk stratification as including two or more individual‐level risk factors beyond age and sex, including phenotypic or genetic factors, in combination to systematically determine elements of the screening programme according to risk. We adopted the Theoretical Framework of Acceptability (TFA) definition of acceptability: ‘A multi‐faceted construct that reflects the extent to which people delivering or receiving a health intervention consider it to be appropriate, based on anticipated or experienced cognitive and emotional responses to the intervention’.^17^ Studies published in peer‐reviewed journals were considered eligible if they were published in English, used primary mixed‐methods, quantitative or qualitative methodology, included views of the public on acceptability and were conducted in the context of risk‐stratified population‐based cancer screening. Studies conducted exclusively with carriers of high‐penetrance genes, such as *BRCA1/2*, were excluded because such individuals are typically managed via surveillance pathways. Similarly, studies conducted in the context of cancer surveillance/monitoring programmes, case finding and non‐risk‐stratified cancer screening were excluded.

The database searches, removal of duplicates and title and abstract review were performed by LT with support from an information specialist. 10% of citations were independently screened by a second reviewer (R. D., A. H. or K. L.) and disagreements were resolved in consensus meetings. Citations eligible for full‐text review were reviewed by two reviewers. Studies excluded by both reviewers were considered ineligible and discrepancies were again resolved via consensus. We screened the reference lists of the eligible citations and two related systematic reviews^12^, ^13^ to identify any papers that were not found in the main database search.

### 2.3 Data extraction and synthesis

Data extraction was conducted by L. T. using a standardized form. A. H. also extracted the results and authors' conclusions for 50% of eligible citations to reduce bias. The Joanna Briggs Institute (JBI) convergent integrated approach was used for data extraction and synthesis as both quantitative and qualitative data address the research question.^18^, ^19^, ^20^ Qualitative data defined as results in the paper of origin were extracted directly using NVivo 12 (QSR International Pty Ltd.; released 2018), including any supplementary files. We transformed quantitative results into qualitative statements via narrative interpretation, retaining numerical results and contextual anchors to maintain the integrity of the findings.^19^ The resulting textual statements were also imported into NVivo 12.

The TFA was used as the coding frame for thematic analysis as it was developed specifically for use in healthcare interventions and enables prospective or anticipated assessment of the intervention where it has not yet been implemented, as in the case of risk stratification.^17^, ^21^, ^22^ Additionally, it considers individuals' cognitive and emotional responses, unlike more process‐driven frameworks.^21^ The TFA includes seven constructs of acceptability, defined here as:

1. Affective Attitude: how people feel about risk‐stratified cancer screening.
2. Burden: perceived amount of effort required to participate.
3. Ethicality: how well risk stratification aligns with individuals' values.
4. Intervention Coherence: how far people understand risk‐based screening and how it works.
5. Opportunity Cost: benefits, resources or principles that must be given up to participate.
6. Perceived Effectiveness: how likely the public feel the intervention is to achieve its goal.
7. Self‐efficacy: how confident the public are about participating in risk‐stratified screening.

Once the data were coded, we reviewed the contents of each high‐level construct and generated themes and sub‐themes by identifying and interpreting areas of similarity across the data. The contents of the sub‐themes were reviewed and summarized to understand areas of convergence, divergence and ambiguity. This process continued iteratively throughout several meetings with the first author (L. T.) and a second researcher (R. D.). Initial coding using the TFA was undertaken independently before researchers came together to revise and finalize the content of the sub‐themes.

### 2.4 Quality assessment

Two reviewers (L. T. and A. H.) independently completed quality assessment for all studies using the Mixed Methods Appraisal Tool (MMAT),^23^ which is appropriate for assessing the quality of qualitative, quantitative and mixed‐methods research. No studies were excluded on the basis of quality.

## 3 RESULTS

### 3.1 Study selection

**Figure 1:**
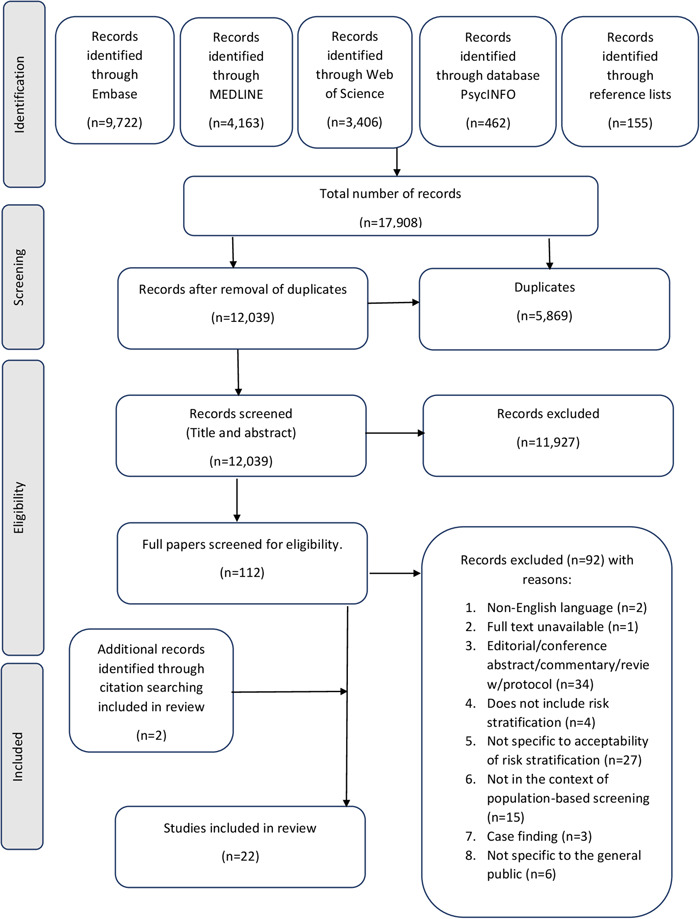
PRISMA flow diagram.

The search generated a total of 12,039 discrete results. We excluded 11,977 citations after title and abstract review, with 96% agreement. A further 92 full texts were excluded. The most common reasons for exclusion were nonempirical articles or not being specific to the acceptability of risk‐stratified screening (Figure 1). Additionally, we identified two citations after searching reference lists. This resulted in 22 papers meeting the eligibility criteria for inclusion.^5^, ^24^, ^25^, ^26^, ^27^, ^28^, ^29^, ^30^, ^31^, ^32^, ^33^, ^34^, ^35^, ^36^, ^37^, ^38^, ^39^, ^40^, ^41^, ^42^, ^43^, ^44^

### 3.2 Study characteristics

The study characteristics and authors' primary conclusions are summarized in Table 1. Most studies considered breast cancer screening (*N* = 15/22, 68%) and^5^, ^25^, ^26^, ^28^, ^29^, ^30^, ^32^, ^33^, ^34^, ^36^, ^37^, ^39^, ^40^, ^41^, ^43^ four focused on ovarian cancer (*N* = 4/22, 18%),^27^, ^38^, ^42^, ^44^ with one study involving kidney, prostate, colorectal and all cancer types.^24^, ^28^, ^31^, ^35^ Participant characteristics varied in terms of sex and age, and two studies specifically considered the views of ethnic minority populations.^33^, ^38^ Sample sizes ranged from eight^44^ to ten thousand^28^ participants, distributed across 9 quantitative (*N* = 9/22, 41%)^5^, ^24^, ^25^, ^26^, ^27^, ^28^, ^29^, ^30^, ^31^ and 13 qualitative studies (*N* = 13/22, 59%).^32^, ^33^, ^34^, ^35^, ^36^, ^37^, ^38^, ^39^, ^40^, ^41^, ^42^, ^43^, ^44^ The majority of the included studies took place in the UK (*N* = 13/22, 59%).^5^, ^24^, ^27^, ^29^, ^30^, ^32^, ^33^, ^36^, ^38^, ^41^, ^42^, ^43^, ^44^

**Table 1:** Summary of included study characteristics and primary conclusions.

Author & year	Setting (country)	Cancer type(s)	Sample size	Age	Sex	Study design	Method of data collection	MMAT result	Author's primary conclusion(s)
Meise et al.^29^	UK	Breast	942	18‐74	F	Quantitative	Home‐based interview survey embedded within ONS survey		Women were positive about adjusting mammography screening in line with personal genetic risk. Effective communication materials should be developed to minimise resistance to reducing screening frequency for those at lower genetic risk.
Koitsalu et al.^28^	Sweden	Breast & prostate	10,000	20‐74	M & F	Quantitative	Online cross‐sectional survey		Risk‐stratified screening has the possibility to be accepted by the general public. Knowledge about interest & acceptability of the prospect of risk‐stratified screening for BC & PC will help when implementing new screening strategies.
Meisel et al.^27^	UK	Ovarian	829	18‐74	F	Quantitative	Home‐based interview survey embedded within ONS survey		Women in the UK are ready to accept the introduction of population‐based genetic testing for personalisation of future OC screening programmes.
Piper et al.^31^	US	Colorectal	1,054	>50	M & F	Quantitative	Mail‐based survey		Many veterans are resistant to stopping low‐value CRC screening & are sceptical about CRC risk & life expectancy predictions, even using risk calculators. Risk stratification may be met with resistance. Future research is needed to understand how to effectively communicate with patients on this topic.
Ghanouni et al.^5^	UK	Breast	933	40‐70	F	Quantitative	Face‐to‐face survey embedded within unrelated survey		Women were generally in favour of risk assessment & more frequent stratified screening. Common barriers included worry & preferring not to know risk status. Letters/emails were preferred for receiving very low risk status & face to face for very high risk. GPs were the preferred sources of information. Participants were relatively unwilling to accept less frequent or no screening for very low risk.
Ghanouni et al.^30^	UK	Breast	698	40‐70	F	Quantitative	Online survey with vignettes		No information on order effect of preferences was observed. Information order did affect risk perceptions. Women who perceive themselves as high risk may be particularly resistant to less frequent screening.
Rainey et al.^26^	Netherlands	Breast	942	50‐75	F	Quantitative	Structured survey presented in two parts		Dutch women were generally in favour of receiving BC risk & personalised screening recommendations. Acceptance depends on assigned risk category – most challenging for low‐risk. Education on the benefits & harms of risk‐based screening is key to acceptability & informed decision‐making.
Mbuya‐Bienge et al.^25^	Canada	Breast	4,293	30‐69	F	Quantitative	Structured online survey		Risk‐stratified BC screening is likely to be supported by Canadian women. Further research should seek to understand why some women are reluctant to provide personal & genetic information for risk assessment & how interactions between different factors shape women's' views.
Usher‐Smith et al.^24^	UK	Kidney	668	45‐79	M & F	Quantitative	Online population‐based survey		Risk stratification of starting age based on phenotypic or genetic risk modelling is acceptable to the majority of individuals & has the potential to increase uptake.
Henneman et al.^40^	Netherlands	Breast	26	42‐73	F	Qualitative	Semi‐structured focus groups		Women currently offered BC screening based on age have a positive attitude towards screening for BC. More research is needed if women in the low‐risk group are to be no longer offered mammography screening.
Meisel et al.^42^	UK	Ovarian	56	Mean 45	M & F	Qualitative	Guided focus groups		The public may be broadly positive about population‐based genetic testing for OC & would support risk‐stratified screening, providing preventive advice was offered alongside the programme.
Rahman et al.^44^	UK	Ovarian	8	25‐58	F	Qualitative	Semi‐structured interviews		Despite variation in age, family history & OC experiences, there was strong consensus that a population‐based risk prediction, stratification & screening program for OC should be introduced. The main anticipated benefit was relief & reassurance for the majority of the population who would be classified at low risk for developing OC.
Hann et al.^38^	UK	Ovarian	62	18+	M & F	Qualitative	Community‐based focus groups		Population‐based risk assessment & stratified management may be acceptable to many SA men & women in the UK. Attitudes towards cancer screening were positive. Views on risk‐reducing surgery were mixed. There is a need for tailored OC awareness campaigns within SA communities.
He et al.^39^	US	Breast	29	40‐74	F	Qualitative	Semi‐structured focus groups and surveys		Some women thought risk‐based screening made sense. Willingness to abandon traditional screening in favour of risk stratification was mixed. Broad acceptability of risk‐based screening will require clearer communication about its rationale & feasibility & consistent messages from HCPs.
Lippey et al.^37^	Australia	Breast	52	48‐72	F	Qualitative	Focus groups		Although risk stratification is a feasible concept to women who are currently engaged in BC screening, participants were particularly concerned about a lower risk cohort being lost to the system, the importance of communication & the motivations behind the change.
Rainey et al.^41^	Netherlands, UK, Sweden	Breast	143	40‐75	F	Qualitative	Focus‐groups with semi‐structured interview guide		Acceptability of risk‐based screening & prevention is mixed. More intensive screening for women above average risk was generally welcomed. Screening pathways for the other risk categories & general prevention strategies were met with some scepticism. Women's perceptions were informed by a lack of knowledge, cultural norms, & common emotional concerns, highlighting the importance of tailored educational materials & risk counselling to aid informed decision making.
Rainey et al.^36^	Netherlands, UK, Sweden	Breast	143	40‐74	F	Qualitative	Semi‐structured focus groups		Existing healthcare policy & care pathways will impact implementation of risk‐based breast screening. Country‐specific assessment, communication protocols & uniform screening recommendations need to be developed. Additional training needed for HCPs.
Woof et al.^33^	UK	Breast	19	47‐73	F	Qualitative	Semi‐structured interviews		Risk stratification was viewed favourably. To avoid exacerbating inequities, information should be provided in multiple languages & modalities & offer the opportunity to speak to a HCP about risk. Completion of personal risk information should be via paper questionnaires, as well as online.
Dunlop et al.^35^	Australia	All types	40	18‐69	M & F	Qualitative	Semi‐structured interviews		Acceptability of risk‐stratified screening for different cancers may be impacted by type of screening test, level of attachment to the screening programme & the type of cancer itself. Acceptance of risk‐stratified screening is high, but most people are unlikely to forgo screening altogether even if at low‐risk.
Kelley‐Jones et al.^43^	UK	Breast	25	40‐70	F	Qualitative	Interviews		There is high, but not universal, acceptability of risk‐stratified BC screening. Support & guidance, tailored to screening values & preferences may be required at all levels of risk.
McWilliams et al.^32^	UK	Breast	23	46‐74	F	Qualitative	Semi‐structured interviews		It is acceptable to receive a low‐risk estimate as part of BC screening, from the perspective of a generally well educated, mainly white British sample. Challenges were highlighted in ensuring informed choice given the introduction of less frequent screening for women at low risk of breast cancer. Any approach where screening is reduced for low‐risk women should be clearly explained & evidence‐based.
Sierra et al.^34^	Australia	Breast	31	21‐70	F	Qualitative	Individual interviews and focus groups		Implementation of a PBCRA is acceptable. Women are particularly concerned with actionability of results & reduced screening, especially for those who view themselves as high risk. It will be important to educate the wider population, both women & HCPs, on BC genetics, risk factors, & determine optimal risk‐category‐specific screening methods. Systems must be in place to deliver genomic information with adequate emotional & psychological support.

Abbreviations: BC, breast cancer; CRC, colorectal cancer; F, female; GP, general practitioner; Green, ‘yes’ for all MMAT domains; HCP, healthcare professional; M, male; MMAT, Mixed Methods Appraisal Tool; NHSBCSP, National Health Service breast cancer screening programme; OC, ovarian cancer; ONS, Office of National Statistics; PBCRA, personalized breast cancer risk assessment; PC, prostate cancer; PRA, personalized risk assessment; Red, ‘no’ for one or more MMAT domains or ‘can't tell’ for two or more MMAT domains; RSBS, risk‐stratified breast screening; SA, South Asian; Yellow, ‘Can't tell’ for one MMAT domain.

### 3.3 Quality assessment

As shown in Supporting Information: Table S2, 10 studies were high quality across all domains (*N* = 10/22, 45%).^27^, ^28^, ^29^, ^32^, ^33^, ^35^, ^37^, ^38^, ^43^, ^44^ The remaining studies were of lower quality, scoring ‘no’ or ‘can't tell’ in at least one domain. The most common lower scoring domains were ‘Is the sample representative of the target population?’ and ‘Is the risk of nonresponse bias low?' for quantitative studies, and ‘Is there coherence between qualitative data sources, collection, analysis, and interpretation?’ for qualitative studies.

### 3.4 Distribution of the evidence across themes

The majority of themes were considered across multiple studies (Supporting Information: Table S3); however, some were contributed to only briefly and ‘The importance of prevention and early detection’ was only considered by three studies.^28^, ^35^, ^44^ The qualitative studies in particular included multiple themes in detail, with four papers contributing to over 10 themes.^32^, ^34^, ^35^, ^43^ The strength of evidence was greatest for ‘The impact of knowing your risk’ as this was covered by 16 studies, 6 of these in detail.^26^, ^32^, ^38^, ^41^, ^42^, ^44^

### 3.5 Affective Attitude (Table 2)

**Table 2:** Summary of themes and illustrative quotations: Affective Attitude.

Themes	Sub‐themes	Illustrative quotes
General attitudes towards risk stratification		The majority of women reported that it is a good or very good idea to use personal information (72.8%) and results from genetic tests (72.8%) to identify women who are at high, average, or low risk of developing breast cancer, and to change how often women are invited for breast screening based on this information (63.5%).^25^
How to communicate risk assessment results	Preference for face‐to‐face delivery of high‐risk estimates	Women had mixed opinions on the acceptability of receiving a personalised risk result via letter. The acceptability of the approach was dependent upon the severity of the result. Women were concerned that a high‐risk result received via letter would cause distress. They felt that without the immediate opportunity to ask questions, women might speculate as to what a high‐risk result would mean for their health. In this instance it was suggested that a face‐to‐face consultation with a healthcare professional would be more suitable:
		*She said someone with a high‐risk, it would be quite scary and they might have so many questions that are buzzing through their head then at that point. So it would be better really for someone in that position who's in a higher risk to be able to be told face‐to‐face*. (Fatima, 60, via interpreter)^33^
	Variation in preferences for risk formatting	Swedish women indicated that they would like to have their risk expressed in a proportion. Additionally, Dutch and Swedish women would like to see their risk represented both in a percentage and visually. Dutch women indicated a preference for recording the risk appointment and both Swedish and Dutch women would like to receive the information in writing to take home.^36^
		…women indicated that it would be useful to receive their personal risk feedback and screening recommendations as ‘a ladder of risk’ as this may reflect how they evaluate their level of risk:
		…*the human psyche does weigh things up and you look at who's above you and below you… placing yourself on a sort of scale*. (17: Occasional screening attendee, 51 years)^43^
	Desire for education, prevention and lifestyle advice	Women were cognisant about how important it would be to communicate any change to the current model of breast screening clearly and concisely to the target population and that education would be a vital component to the process.
		*So does that mean maybe an education process? Not just a testing process, and filling in a form and having a little chat, but an education process, so you understand all of those factors better… because you've had it all explained to you*. (Participant 4, Focus group 3).^37^
The impact of knowing your risk	The desire to know your risk	Dutch and Swedish women were generally positive about receiving breast cancer risk feedback. None of the British women expressed regret about finding out their risk. Women in all three countries emphasized, however, that participation should be optional, offering screening according to current country guidelines to women who do not want to adopt this approach.^41^
	Advantages of knowing your risk	Based on our presentation of genetic risk and genetic testing for OC, in discussion most participants initially expressed positive views. They felt they would benefit from knowing if they were at increased risk because they could take steps to manage their individual risk.
		*So you are aware of it, and you know how to prevent it, getting information, what are the risks, and how to do your daily activity, your daily lifestyle, maybe that can change…* (FG2, woman, London)^38^
	Disadvantages of knowing your risk	The concept of an intermediate risk group was met with some hesitancy by the participants. A number of women were unsure of what benefit having this information would provide. The perception was that individuals in this risk category may be concerned at being at increased risk compared to the low risk group, but without being offered the surveillance or prevention options available to those in the high risk group.^44^

*Note*: Quotes in italics represent those from participants. Quotes that are not in italics represent those of the author. Participant characteristics have been included where available in the original paper.

Abbreviations: FG, focus group; O C, ovarian cancer.

#### 3.5.1 General attitudes towards risk stratification

Risk stratification was considered generally acceptable,^5^, ^24^, ^25^, ^27^, ^29^, ^31^, ^44^ with over 70% of women reporting that they would take up risk‐stratified breast screening if offered.^5^ A comprehensive lifestyle or genetic risk score was perceived as more acceptable than less comprehensive models,^24^ and the inclusion of genetic variables was largely viewed with optimism.^29^ However, the prospect of a risk‐stratified cancer screening programme was met with more caution than risk assessment alone.^43^

#### 3.5.2 How to communicate risk assessment results

Participants acknowledged that mass communication is difficult because the screening population is large and diverse. However, the higher one's cancer risk, the greater the perceived need for individual consultation, because receiving a high‐risk estimate could generate anxiety and questions.^5^, ^26^, ^28^, ^33^, ^36^, ^44^ A trusted HCP, such as a GP or specialist nurse, was preferred to deliver results and address concerns.^5^, ^26^, ^28^, ^33^, ^36^ Receiving average or below‐average risk estimates via letter was considered appropriate.^5^, ^28^, ^33^, ^36^

Opinions on presenting risk‐based information varied and participants proposed multiple formats to maximize understanding, including visual aids/diagrams, relative and absolute risk, percentage risk and age‐related risk.^34^, ^36^, ^44^ Provision of written risk information to take away and reflect upon was encouraged.^36^ Some participants preferred comparative risk information, where individual risk is provided in the wider context of a ‘ladder of risk’.^44^ Delivering results in this way helped to put them into perspective, enabling participants to weigh up and contextualize personal risk.^27^, ^43^

Many participants wanted educational and preventative advice, describing knowledge as power.^35^, ^37^, ^41^, ^42^, ^44^ Public education about changes to the screening programme, risk factors, risk reduction and cancer itself was of high importance.^37^, ^41^, ^42^, ^44^

#### 3.5.3 The impact of knowing your risk

The desire for personalized risk information was high; however, it was considered essential that participation in risk assessment should not be mandated.^41^ Discussion around the impact of knowing your risk tended to focus on potential advantages. Many participants found knowledge of personalized cancer risk to be empowering, stating that it would help raise awareness and enable proactive behaviour.^33^, ^38^, ^41^, ^42^ A low‐risk result could provide reassurance and relief^32^, ^35^, ^42^, ^44^ and high‐risk individuals could benefit from the ‘safety net’ of increased surveillance or early intervention.^38^, ^43^, ^44^ Knowledge of personal risk could assist informed decisions about hormone replacement therapy, risk‐reducing medication or prophylactic surgery, lifestyle and future screening attendance.^24^, ^26^, ^32^, ^40^, ^43^

The potential disadvantages of receiving a risk estimate were also considered. One area of concern was the introduction of an intermediate‐risk category. Some participants felt that falling outside of the low‐risk category, yet not receiving the same benefits as a high‐risk group may serve to increase intermediate‐risk individuals' anxiety without any obvious advantage.^44^ Similarly, if perceived cancer risk does not correlate with risk assessment results, then the resultant screening recommendations were deemed less acceptable.^41^ A low‐risk result was believed not to justify ‘complacency’ and it was described as ‘cruel’ to deliver risk feedback without providing advice about lowering that risk.^42^

### 3.6 Burden (Table 3)

**Table 3:** Summary of themes and illustrative quotations: Burden.

Themes	Sub‐themes	Quotes
Barriers to accessibility	General concerns about accessibility	Participants, particularly those at general population risk, stated that people are more likely to participate if the PBCRA is convenient to access. Factors influencing convenience included: cost, location, effort, type of test (blood vs. saliva, separate test vs. added to standard blood work), and appointment type.
		*I think the easier you make it you could get to, in any way that you make it easy to do. Easy to access, easy to send off, cheaper, you know all of those things are going to get more people involved*. (49 years, general population)^34^
	Culture‐specific concerns about accessibility	Women acknowledged that if materials could not be automatically provided in their spoken language, the option to request translated versions would be beneficial. However, providing translations would not make materials accessible for all, with many of the women identifying that not all women in their communities are literate in the languages they speak.^33^
Emotional or psychological burden		All women agreed that the PBCRA has the potential to cause anxiety or stress, especially when identifying women at above‐average BC risk. The additional knowledge may affect an individual's peace of mind or create feelings of panic, helplessness, and the perception that cancer is inevitable. Some women feel it may be better not to know their cancer risk, however, this is dependent on their personality as some individuals are more prone to anxiety.
		*Just the anxiety if you know, of living with that. Some people get very stressed like me (laughs)*. (58 years, general population)^34^

*Note*: Quotes in italics represent those from participants. Quotes that are not in italics represent those of the author. Participant characteristics have been included where available in the original paper.

Abbreviations: BC, breast cancer; PBCRA, personalized breast cancer risk assessment.

#### 3.6.1 Barriers to accessibility

Ease of access was considered a predictor of participation in risk assessment and in a risk‐based screening programme.^32^, ^33^, ^34^, ^36^, ^38^, ^39^ Some participants found it difficult to provide risk information^32^ and felt that completing a risk assessment or providing risk results online would represent a barrier to accessibility, particularly for older generations.^33^, ^38^ Financial burden, location, perceived effort and the type of screening test offered contributed to the idea of convenience.^34^

Two studies explicitly examined cultural barriers and found that ethnic minority groups were likely to experience language barriers on top of computer literacy issues.^33^, ^38^ The ideal solution was to receive a letter containing all information in their first language or have the option to request translations. Nevertheless, not all individuals in minority communities are literate in the languages that they speak, which may exacerbate accessibility issues for written communication.^33^ Additionally, a high‐risk estimate carried the potential to impact marriage prospects for younger women and the perceived acceptability of risk‐reducing surgery may be influenced by the strong cultural importance of having.^38^

#### 3.6.2 Emotional or psychological burden

The psychological consequences of risk‐informed screening^5^, ^28^, ^32^, ^34^, ^35^, ^38^, ^40^, ^41^, ^42^, ^43^, ^44^ overlapped somewhat with the ‘impact of knowing your risk’. The primary concern was the potential for increased stress and anxiety. Participants recognized that although anxiety depends upon individual disposition or personality, receiving a personalized risk estimate may generate negative emotions.^32^ Potential for worry was cited as the most common reason not to participate in a breast cancer risk assessment (14%).^5^

The majority of participants felt that psychological consequences would be greatest among high‐risk cohorts, who would experience the emotional burden and logistical inconvenience of increased screening, stating that ‘A high‐risk result almost feels like a diagnosis’.^35^, ^38^, ^41^, ^43^ Concerns over the intrusive nature of increased screening were identified, describing it as a ‘shadow’ and something you carry for the rest of your life.^35^, ^40^, ^43^ In light of these issues, participants suggested the option for HCPs to be notified of risk estimates without the public needing to find out themselves.^42^

### 3.7 Ethicality (Table 4)

**Table 4:** Summary of themes and illustrative quotations: Ethicality.

Themes	Sub‐themes	Quotes
The importance of prevention and early detection		Overwhelmingly, participants felt that early detection of cancer in the broad sense was important and expressed faith in cancer screening to prevent and detect cancer early. Many described screening as a proactive measure in the ‘battle’ against cancer, often providing a definitive answer.
		*Prevention or recognising and identifying problems early on is obviously far more important than waiting until there are problems. So yeah, I'm very much for prevention or early detection*. (Female, 46 years, control group)^35^
Is risk stratification fair?		In one account, a woman viewed it as unfair to not be allowed to make her own decision despite acknowledging that the cervical screening interval changes depending on age. However, others wanted to be told how frequently they should attend mammograms as would feel unable to decide on their own.^32^
Cost as the motivation behind transitioning to risk‐stratified screening		Suspicion about motivation for change to the current model of screening. In each group there was at least 1 participant who expressed concern about the motivation for this potential change with suspicion that the change was being driven to save money at the cost of individual health.
		*We're so used to… hearing the politicians going on about the money and we're going to do this because it's better for you… It's got nothing to do with the money. Which we all know is not true*. (Participant 6, Focus group 2)^37^

*Note*: Quotes in italics represent those from participants. Quotes that are not in italics represent those of the author. Participant characteristics have been included where available in the original paper.

#### 3.7.1 The importance of prevention and early detection

Early detection and prevention were valued attributes of cancer screening and participants derived a sense of proactivity from engaging in screening.^35^ Participants felt strongly that all cancers should be treated equally. Nonetheless, breast cancer was disproportionately remarked upon, possibly in reference to well‐established screening practices.^35^ 97% of women would definitely or maybe attend mammography screening regardless of screening frequency, highlighting its perceived importance.^28^

#### 3.7.2 Is risk stratification fair?

Several aspects of risk‐stratified cancer screening were discussed as having the propensity to be unfair or discriminatory. One area of contention was prioritizing older people as they are more at risk of developing cancer and have contributed to (national) health insurance compared with younger people who may have more to lose.^32^ One individual found it unfair not to be able to choose their own cervical cancer screening intervals, whereas others felt ill‐equipped to make independent decisions.^32^

Participants felt strongly that the same risk assessment should be offered to all in the interest of fairness and accuracy.^36^ However, some inconclusively queried how to fairly categorize people who declined to provide personal risk information.^43^

#### 3.7.3 Cost as the motivation behind transitioning to risk‐stratified screening

Participants were suspicious that cost‐cutting was driving the shift towards risk stratification at the detriment of health and in the absence of a suitable evidence base,^32^, ^37^, ^39^, ^41^ although they did consider that tailored screening could optimize the use of national funding.^32^ The need to communicate the rationale in a clear and considered way to avoid appearing at odds with initiatives aimed at promoting screening uptake was stressed.^32^

### 3.8 Intervention Coherence (Table 5)

**Table 5:** Summary of themes and illustrative quotations: Intervention Coherence.

Themes	Sub‐themes	Quotes
Risk stratification is logical in principle		Despite their concerns and conditions, most participants perceived RSBS to be an improvement over the current age‐based breast screening:
		*It really makes sense with all that added information and you can only get better treatment… the old system seems a bit dated* (11: Pre‐eligible, 46 years).
		One respondent thought that this ‘old system’, i.e., age‐based breast screening now appeared to be lacking:
		*I don't see where those high‐risk groups, especially, because that's where the focus needs to be, they're just flowing through at the same rate and that could be where there's a lot of issues* (18: Pre‐eligible, 40 years).^43^
Understanding the evidence for risk stratification	Variation in understanding of the evidence	Women seemed to understand the idea of combining individual risk measures to compute an overall level of personal risk and appreciated the rationale for this in terms of their perceived limitations of a ‘one size fits all' screening model:
		*So just because you're female and got boobs doesn't automatically mean you're at risk* (16: Regular screening attendee, 67 years).^43^
		Nearly half of the women were confused about current mammography guidelines, including when to start or how often to go. Few understood why screening guidelines changed:
		*You know it's always changing and we really don't know what they base it on or what their group is that they're making those decisions*.^39^
	Specific considerations regarding genetics	Risk‐stratified screening after risk assessment incorporating genetic testing was received with enthusiasm throughout. In fact, in some groups the idea was brought up before the moderator introduced it:
		*Why not just have an umbrella genetic screening to show prevalence and maybe initially to start with one particular age group that has propensity to acquire cancer of some description at a certain stage in life?* ^42^
		There was concern about the overemphasis on genetics within the risk algorithm and apprehension that the more commonly accepted risk factors, such as family history, would be overlooked.
		*Too much emphasis on genetics. Really it's only called for if there's an obvious family lineage in my opinion. And even then, you'd probably just confirm what you already know* (Participant 9, Focus group 2).
		*Genetics isn't predestination* (Participant 1, Focus group 1).^37^

*Note*: Quotes in italics represent those from participants. Quotes that are not in italics represent those of the author. Participant characteristics have been included where available in the original paper.

Abbreviations: HRT, hormone replacement therapy; RSBS, risk‐stratified breast screening.

#### 3.8.1 Risk stratification is logical in principle

Compared with current screening practices, risk‐stratified screening was considered more efficient and cost‐effective, making for a better and more logical screening programme overall.^34^, ^40^, ^43^ For some, it was a ‘natural progression’ to modernize longstanding screening practices.^35^, ^37^, ^43^ Additionally, participants felt that risk stratification could increase early detection, reducing the need for treatment later.^32^ Even those who had reservations about risk‐stratified breast screening still considered it a positive improvement to the current programme.^43^ Despite this, several participants who initially found risk stratification to be a logical concept found the idea of de‐intensifying screening for low‐risk cohorts to be illogical and hard to believe.^35^

#### 3.8.2 Understanding the evidence

Many participants demonstrated poor understanding about current screening practices and did not understand the harms or resource limitations that screening guidelines are based on, making it difficult for them to suggest what features a risk‐stratified screening programme should have.^32^, ^39^, ^43^ For example, participants found weighing up whether to accept diverse screening intervals challenging because they did not understand screening harms and benefits.^32^, ^43^ Even after being informed about a genetic risk assessment, participants struggled to comprehend that personalized risk assessment is not a diagnostic test for cancer.^38^

Understanding of risk‐stratified screening concepts was high once they had been explained;^37^, ^43^ nevertheless, there were areas requiring more evidence to support decision‐making. Some questioned how different risk factors were weighted and the accuracy of risk estimates.^32^, ^39^, ^41^ The belief that personalized screening would be dictated by scientific evidence was tempered by concerns that risk prediction was insufficiently linked to cancer development.^41^ A key concern was the unstable nature of included variables, notably lifestyle factors.^37^, ^40^ As such, periodic reassessment was desired to account for the impact of these changes.^37^, ^41^, ^43^

Genetic risk in particular was explored in four studies.^34^, ^37^, ^40^, ^42^ The appropriate age to offer genetic risk assessment was debated, considering factors such as consent, maturity/responsibility and the value of receiving genetic information at a given time.^34^, ^40^ Most participants felt that genetic testing should be offered to young adults to identify high‐risk individuals sufficiently early.^34^, ^40^ Concerns were raised, however, about a perceived overemphasis on genetics in two studies suggesting that other risk factors, predominantly lifestyle characteristics, could be overlooked or neglected.^37^, ^40^ Others suggested inclusion of genetic information before being prompted and anticipated its utility across a spectrum of cancer types.^40^, ^42^

### 3.9 Opportunity Cost (Table 6)

**Table 6:** Summary of themes and illustrative quotations: Opportunity Cost.

Themes	Sub‐themes	Quotes
Cost of screening and of lifestyle changes		Some Dutch and British women also mentioned potential costs associated with diet and lifestyle changes. They feared that the principle of solidarity in healthcare finance and delivery will be hindered.^36^
		Several women believed that it is not financially feasible if everyone gets a DNA test. For example, this woman had reservations concerning the new developments because of the financial costs, and argued that other projects deserved more financial support than a screening programme that included genetic testing:
		*Of course, I think I'm supportive, but be real. You can only spend your money once. Is it wise to spend our money on this? There may be other health care projects that need financial support more than this one* (48 years).^40^
Data security, privacy & the potential for discrimination		However, most participants mentioned the importance of confidentiality and privacy despite being happy for this information to determine their risk.
		*…the individual getting that information I'm okay with, it's when it starts going into a big bucket‐ o‐ data that I get concerned because once again, it's about the controls around that*. (Male, 42 years, average‐ risk)^35^

*Note*: Quotes in italics represent those from participants. Quotes that are not in italics represent those of the author. Participant characteristics have been included where available in the original paper.

#### 3.9.1 Cost of screening and of lifestyle changes

Several participants voiced reservations about potential opportunity costs, particularly the financial impact of population‐based genetic testing, believing that there are other areas of healthcare where these funds may be better spent.^40^ Additionally, the potential cost to individuals of making changes to diet and lifestyle could impact perceived fairness.^36^ For some participants, the costs of risk‐stratified screening were seldom discussed.^38^

#### 3.9.2 Data security, privacy and the potential for discrimination

In accordance with Intervention Coherence, many participants had opinions about implementing a programme using genetic risk specifically. Although some participants were satisfied with the use of genetic information to inform risk modelling, they had reservations about confidentiality and privacy to avoid unlawful or inappropriate usage.^35^, ^37^ Another central concern was how genetic information might impact insurance and whether employers may discriminate based on what was described as a ‘genetic passport’.^34^, ^37^, ^40^ Others felt unconcerned by the use of genetic information in insurance matters, either not mentioning it or actively supporting its use.^37^, ^42^

### 3.10 Perceived Effectiveness (Table 7)

**Table 7:** Summary of themes and illustrative quotations: Perceived Effectiveness.

Themes	Sub‐themes	Quotes
Considerations for people at low risk		Despite being largely accepting of the possibility of less frequent breast screening, women viewed the current 3‐yearly programme favourably overall. Some women acknowledged that interval cancers and false positives already exist within 3‐yearly screening however, concerns about having fewer mammograms related to a loss of safety that breast cancer will be detected quickly.^32^
		‘Overall acceptors’ had mixed responses to the prospect of foregoing screening if at very low risk. Although some considered it reasonable and rational, acceptability was conditional on clearly sign‐posted self‐referral pathways for screening. There were also indications of an endowment effect with some ‘overall acceptors’ who were regular attenders suggesting that although they would find it personally unacceptable, they could see future generations of women thinking otherwise.^43^
The impact of HCP involvement on implementation and delivery		Another concern for women at moderate to high risk was the knowledge of their GPs. The majority of British FGD participants indicated that their GP was insufficiently informed about tamoxifen/raloxifene and their usage as preventative medication. Consequently, a majority of GPs refused to prescribe the risk‐reducing medication, referring women back to the research team.^36^
		All women preferred a test endorsed and performed by people or institutions they were
		familiar and comfortable with, such as their physicians, the government, and BreastScreen Australia. Women report they want to feel safe and have access to appropriate resources if needed, and want to receive consistent advice from all their healthcare providers.^34^
The impact of risk assessment on wider outcomes		The majority of women said they would have a PBCRA if offered, without any additional information needed. However, women felt that successful implementation of the PBCRA relies on the availability of appropriate risk management strategies and adequate connection to health and support systems. Furthermore, women found the prospect of the test more appealing when more management and screening options were made available to them.^34^

*Note*: Quotes in italics represent those from participants. Quotes that are not in italics represent those of the author. Participant characteristics have been included where available in the original paper.

Abbreviations: FGD, focus group discussion; GP, general practitioner; HCP, healthcare professional; PBCRA, personalized breast cancer risk assessment.

#### 3.10.1 Considerations for people at low risk

Considerations around low‐risk cohorts were debated more widely than increased screening for high‐risk ones.^25^, ^28^, ^31^, ^32^, ^34^, ^35^, ^37^, ^39^, ^40^, ^42^, ^43^ Some participants reported positive or neutral reactions to reduced screening or found extended screening intervals strongly unacceptable.^32^, ^37^, ^42^ In some cases, the benefits for low‐risk individuals were well understood, including better use of funds and resources and reduced harm.^35^, ^39^, ^43^ However, fears were expressed about safety, including possible interval cancers and late‐stage diagnoses, meaning that the value of screening was felt to outweigh the risk of harm.^32^, ^37^, ^39^, ^43^ For breast cancer specifically, menopause was of concern due to associated hormonal changes and some women in this age bracket found it especially challenging to accept reducing screening.^43^

Familiarity with screening and being a regular attender might affect perceptions of reduced screening.^34^, ^37^, ^43^ Many participants derived reassurance and security from current screening practices, making it harder for them to accept reduced screening.^34^, ^37^ However, it was acknowledged that there may be an endowment effect, with younger generations who are not currently eligible finding it more acceptable to screen low‐risk groups less intensively.^34^, ^37^, ^43^ Furthermore, some individuals felt more willing to reduce or stop screening if at low risk in instances where the screening tests were unpleasant or invasive, namely, colonoscopy and cervical smear.^35^ Higher trust in the HCP recommending cessation was positively associated with willingness to stop screening (odds ratio: 1.19; 95% confidence interval: 1.07–1.32).^31^

Participants acknowledged that a low risk of developing cancer does not mean no risk and therefore preferred reducing screening frequency rather than cessation, which was largely viewed as unacceptable.^40^ Those who found reduced screening unacceptable wanted more reassurance, either physical examination conducted by a HCP or supplementary self‐funded screening.^37^, ^43^ For some, the acceptability of de‐escalated screening relied on clear evidence‐based communication from the healthcare service.^32^, ^43^

#### 3.10.2 The impact of HCP involvement on implementation and delivery

As mentioned in previous themes, participants felt that trusted organizations or individuals should endorse and deliver risk assessment and that confidence in HCPs could impact believability of risk‐stratified screening advice and risk‐reducing interventions.^34^, ^35^, ^41^ Although professional endorsement was seen as essential, several participants were sceptical about the ability of their HCP to deliver this, believing that GPs lack the appropriate knowledge to discuss risk‐reducing interventions with moderate‐ to high‐risk patients.^36^, ^44^ Another caveat to effective implementation was contradicting advice from different HCPs.^34^, ^41^ As such, participants identified a need for standardization and implementation of specific pathways to improve consistency between primary and secondary care.^36^

#### 3.10.3 The impact of risk assessment on wider outcomes

Participants were largely positive about the impact of personalized risk assessment but most agreed that appropriate risk‐management infrastructure and screening options should be available after risk assessment.^34^ Risk assessment was more acceptable when participants knew that there would be risk‐based screening, management and support options provided afterwards.^34^

### 3.11 Self‐efficacy (Table 8)

**Table 8:** Summary of themes and illustrative quotations: Self‐efficacy.

Themes	Sub‐themes	Quotes
Feelings of personal responsibility		Although women in all three countries generally welcomed preventative options to manage their risk, they also mentioned the potential for stigma and guilt, e.g.
		*It puts a lot of responsibility for health on women and not everyone is equally capable of maintaining a healthy lifestyle; financially or intellectually. It can't become a woman's own fault if she develops breast cancer (Dutch participant)*.^41^
A need for help and guidance from HCPs		When asked how they would decide whether to opt for longer screening intervals, women identified the limits of ensuring informed choice within screening as felt unaccustomed to deliberate about how much they interact with such programmes. This led women to expect that guidance from healthcare professionals in the field would be provided.^32^
Willingness to participate in risk assessment		Most women were prepared to have a mammogram (96.2%), complete a questionnaire (95.9%), and provide a blood sample (97.6%) for breast cancer risk assessment.^26^
		A majority of the respondents answered being comfortable in conveying their personal information (63%, *n* = 1788) as well as their genetic information (70%, *n* = 1981) to the healthcare workers to assess their cancer risk. However, young respondents appeared to trust the health care more than older respondents (66% vs. 60%, *p* < .001) when it comes to providing personal information whereas older respondents were more often answering that they neither agree nor disagree (32% vs. 23%, *p* < .001).^28^

*Note*: Quotes in italics represent those from participants. Quotes that are not in italics represent those of the author. Participant characteristics have been included where available in the original paper.

Abbreviation: HCP, healthcare professional.

#### 3.11.1 Feelings of personal responsibility

Personal responsibility was most salient in the context of reducing screening opportunities for low‐risk cohorts.^32^, ^34^, ^35^, ^41^, ^42^, ^43^, ^44^ This scenario was felt to place personal responsibility on people to mitigate their risk^32^, ^34^, ^41^, ^43^; therefore, participants needed to know what symptoms to look out for and when to seek medical attention, labelling this increased responsibility as a ‘burden’.^34^, ^43^, ^44^ Although some individuals believed screening decision‐making to be a personal responsibility, others considered a shared decision‐making process with HCPs and family.^41^ Some recognized that not everyone has the same capacity to act upon risk information and felt this could increase guilt and stigma.^41^ Women, more than men, felt a responsibility to be cognisant of their genetic risk for the sake of their offspring.^42^ Conversely, personal responsibility was felt to potentially exacerbate complacency among people who already take little responsibility for their own health.^35^

Melanoma was explored as conceptually distinct from other cancer types due to visibility. Some participants felt that this would make it easier for people to take responsibility and that no screening for low‐risk groups may be more acceptable in this instance. Existing attenders at melanoma screening felt more resistance towards foregoing screening and less confident in their ability to self‐screen.^35^

#### 3.11.2 A need for help and guidance from HCPs

Some participants felt unfamiliar with screening principles and statistics, such as positive/negative predictive values, and consequently recognized the limits of informed choice about participating in risk‐stratified screening. As such, they expected support and guidance from an appropriate professional.^32^ As described above, the provision of advice and reassurance from experts is fundamental to the acceptability of stopping screening after a low‐risk estimate.^35^, ^43^ Where self‐efficacy in checking for symptoms was lacking, participants preferred to request a physical examination from their HCP.^43^ Training or advice in self‐examination for low‐risk groups would enable them to remain vigilant.^43^ A successful programme should provide information about cancer and associated symptoms if participants are expected to make informed decisions about participating in risk assessment.^44^

#### 3.11.3 Willingness to participate in risk assessment

Willingness to complete a questionnaire, including information about reproductive and family history, provide a blood or saliva sample and have a mammogram as part of a breast cancer risk assessment, was high.^25^, ^26^, ^36^, ^43^ Participants with high levels of cancer worry were more willing to undergo risk assessment than individuals reporting that they never worried about cancer (odds ratio; 1.89, *p* = .031).^5^ Willingness to provide genetic information varied, but 70% of participants felt comfortable providing such information, compared to only 63% who were willing to provide phenotypic data.^28^

## 4 DISCUSSION

Our findings suggest that risk stratification within population‐based screening programmes is likely to be acceptable to the public, who considered it to be logical and of wider benefit than existing programmes. The participants across the included studies have, however, raised a number of potential concerns and highlighted key aspects that will be important for implementation and communication of risk stratification. Based on these, we have developed recommendations for future implementation of risk stratification (Table 9). These can be grouped into four key areas.

**Table 9:** Recommendations for communication and implementation of risk‐stratified cancer screening.

Area of consideration	Recommendation	Details
Communication preferences	Personalized risk estimates should be presented in an actionable way and be provided in a variety of formats to maximize understanding.	Risk information should be presented in the context of future actions, including implications for screening, risk‐reducing medications or surgery and lifestyle changes.
		Risk‐adapted screening strategies should be presented in the context of the evidence that supports them to alleviate difficulty in understanding the harms and benefits.
		Risk information should be provided in varied formats including numerical, gist‐based, diagrammatic and comparative expressions of risk to maximize understanding and should be provided in a written format to aid in shared decision‐making and personal reflection.
	High‐risk estimates should be communicated face to face.	Above average risk estimates should be communicated face to face by an appropriate HCP, such as a GP or specialist nurse.
		Average and below average risk estimates should be communicated via letter, but patients should still have the ability to contact a HCP if they have queries or require advice.
	National communication should avoid appearing at odds with existing screening campaigns and be cautious of the motivation behind the transition.	National‐level communication about risk stratification should avoid contradicting existing public health campaigns aimed at increasing uptake of cancer screening.
		National‐level communication should focus on promoting the benefits of risk stratification to individual health and be cautious about presenting cost and resource limitations as the primary motive behind this transition.
Information needs	Information about the rationale of any risk‐stratified screening programme, the risk factors considered and how to reduce cancer risk should be provided.	Public desire for information is high. Information detailing the rationale behind risk‐stratified screening, key risk factors used in modelling, options for risk management and advice on lowering risk via lifestyle changes should be available.
		Desire for education about cancer and preventative advice is also high, particularly among minority communities, where such knowledge may currently be low.
	Information about self‐checking for cancer symptoms and reassurance to seek help when needed should be publicly available.	Individuals may experience feelings of increased responsibility after receiving a risk estimate and/or reduced screening opportunities.
		Information on checking for cancer symptoms should be publicly available and low‐risk individuals in particular should be reassured about the need to seek help from a HCP when necessary.
	The public require reassurance around the use of genetic data.	Incorporation of genetic data into risk modelling should be conveyed tactfully, acknowledging public concerns regarding data security and discrimination.
		The link between genetic risk factors and cancer development should be evidenced.
Barriers to accessibility	Screening‐related resources should be made available online and on paper and in other languages.	Use of the Internet represents a barrier, particularly for older individuals, so risk assessment and delivery of risk results should also be possible using written materials.
		All information relating to a risk‐stratified screening programme should be available in other languages in the first instance or upon request.
		HCPs should be sensitive to possible cultural implications of receiving a high‐risk estimate on factors such as marriage and childbearing.
	Providing risk information should not be mandated.	Completing risk assessment should not be mandatory, but there is a need to decide how to treat people with missing risk data.
The role of HCPs	Evidence for the safety of reduced screening should be communicated clearly by trusted HCPs and healthcare organizations.	Cessation of screening for low‐risk cohorts is likely to be unacceptable to the general public.
		The safety of de‐intensifying screening should be evidenced to address fears about interval cancers and late‐stage diagnoses.
		Information related to reducing screening should be conveyed clearly and mindfully by trusted HCPs and healthcare organizations as a facilitator of acceptability.
		Individuals who are familiar with current screening practices and women in a menopausal age bracket may require more support from HCPs to overcome resistance to reduced screening.
	HCPs should have appropriate knowledge and training to facilitate informed decision‐making with patients.	Patients may have insufficient understanding of screening principles to make informed decisions so HCPs should be able to provide reliable advice and support as part of shared decision‐making.
		PCPs in particular should be equipped with knowledge and resources to support patients to overcome negative perceptions of primary care.
		Information provided by HCPs should be uniform and congruent across primary care, secondary care and national policies.
		Specialist psychological support should be offered for patients at very high risk.

Abbreviations: GP, general practitioner; HCP, healthcare professional.

The first of these relates to communicating personalized cancer risk prediction information in an actionable way. We have made the following recommendations: ensuring that personalized risk information is appropriately formatted, that high‐risk results are conveyed by a trusted HCP and that national‐level communication is mindful of public preferences.

The second key area includes three recommendations for public information needs: provision of information about a risk‐stratified programme, reassurance around genetics and safeguarding low‐risk groups. A challenge in addressing these requirements is the considerable variation in individual needs and preferences. As such, we suggest giving multiple options where possible and empowering people to speak to a HCP.

The third area relates to mitigating barriers to accessibility, such as computer literacy and language barriers. Although these barriers do not apply exclusively to risk‐stratified screening, participants felt that care should be taken not to exacerbate existing inequalities. Also, risk assessment should not be mandated but future research will be required to decide how to invite participants with missing data.

Our findings show that trust in those endorsing and delivering risk stratification has implications for acceptability. The final key area includes two recommendations that encourage HCPs to support reduced screening for low‐risk cohorts by evidencing safety in a clear and transparent manner.

### 4.1 Comparison with other literature

These findings are consistent with the views of HCPs and wider stakeholders reported in our parallel systematic review.^14^ Both members of the public and HCPs were optimistic about risk stratification, with concerns often relating to reducing screening among low‐risk individuals. Although communication requirements and the role of HCPs were identified by both groups, the public had more defined opinions about specific communication modalities and the value of trust while HCPs, notably in primary care, highlighted their need for training in managing and communicating with patients at different risk levels. Conversely, resource limitations were more salient to HCPs, and potential barriers to accessibility and the responsibility of seeking help when needed were primarily considered by the public.

Another systematic review considered public perceptions of risk‐stratified breast screening and primary prevention strategies.^12^ Although not all of the studies included by Rainey et al. were eligible for this review, we included a further 16 papers, 8 studying cancers other than breast. Both reviews found that receiving risk information was considered empowering and participants preferred both web‐based and written screening materials. However, our review also identified the need for face‐to‐face delivery of high‐risk results, barriers for minority groups including language and cultural sensitivity and the role of self‐efficacy after reduced screening as specific preferences for risk communication.

The importance of risk communication and education has been well documented in related literature considering communicating changes to cancer screening programmes and public health policies. As in our review, understanding personalized risk has been proven to be challenging for lay individuals, particularly mathematical concepts, the harms and benefits of screening and where the provision of a risk result is not aligned with an individual's beliefs about risk.^45^, ^46^, ^47^, ^48^

Communication as a facilitator of public acceptability has been further exemplified by reactions to recent changes to cervical screening intervals in the UK where the public felt inadequately informed of the evidence behind interval changes.^49^, ^50^ In line with the concerns raised in our review, women with a greater understanding of the rationale behind extending the screening interval were more accepting of policy changes.^49^ The impact of inadequate communication and education on widening inequalities was of concern in our review and the reality of this has been demonstrated by cervical screening changes, as less educated women demonstrated poorer understanding, indicating that inferior communication can exacerbate existing inequalities.^50^, ^51^ Overall, this suggests that future communication of changes to cancer screening programmes should provide transparent evidence and reassurance in public communications.

### 4.2 Strengths and limitations

Our use of the TFA strengthened this study by providing structure and ensured that key components of acceptability were not overlooked.^17^ However, some findings covered several of the constructs, such as those considering Affective Attitude and Ethicality, making it challenging to disentangle some themes. The relevance and specificity of included papers were improved by the adoption of transparent and pre‐established definitions for acceptability and risk stratification. Use of a recognized approach to conducting mixed‐methods systematic reviews and the inclusion of quantitative and qualitative studies contributed to comprehensive findings. Finally, this review includes views on acceptability of risk‐stratified screening across many different cancer types, including existing and prospective screening programmes, thus increasing the findings' utility and applicability. However, the majority of studies were conducted in the context of breast cancer, suggesting that not all findings will be generalizable across the spectrum of cancer types and indicating a need for more evidence pertaining to other types of cancer in future.

All included studies were conducted in high‐income settings with well‐established cancer screening programmes, meaning that there is an absence of evidence on acceptability among the public in lower‐income countries who may be less familiar with routine screening. Additionally, the strength of the evidence could have been influenced by the diverse aims of the included studies. A further limitation is the potential for publication bias due to exclusion of unpublished literature, although a pilot search did not identify any relevant unpublished studies or grey literature suggesting that the risk of missing such data is low. A community jury study exploring the public acceptability of risk‐stratified eligibility criteria for cancer screening has been published since this search was conducted.^52^ Participant opinions on which factors to include in a risk model varied and concerns were raised over screening reduction and/or cessation and ensuring fairness, which is congruent with the results presented here. Similarly, transparent communication of the evidence behind risk stratification and the importance of education were recognized as integral components of public acceptability.

## 5 CONCLUSIONS

Risk stratification of population‐based cancer screening programmes is perceived favourably by the general public taking part in the included studies. Key considerations for implementation include actionability of risk results, delivery of public information about risk‐stratified screening, mitigating barriers to accessibility and defining roles of clinicians in supporting patients, particularly low‐risk groups.

## AUTHOR CONTRIBUTIONS

Lily C. Taylor conceptualized and wrote the protocol, performed the search, citation screening, data extraction, quality assessment and analysis and wrote the manuscript. Alison Hutchinson contributed to citation screening and quality assessment, and critically reviewed the manuscript. Katie Law contributed to citation screening and critically reviewed the manuscript. Veeraj Shah critically reviewed the protocol and the manuscript. Juliet A. Usher‐Smith contributed to the conceptualization of the protocol, analysis, and data interpretation, and critically reviewed the manuscript. Rebecca A. Dennison contributed to the conceptualization of the protocol, citation screening, quality assessment and analysis and data interpretation, and critically reviewed the manuscript. All authors reviewed and approved the final manuscript.

## CONFLICT OF INTEREST STATEMENT

All authors have no conflicts of interest with respect to the research, authorship, and/or publication of this article.

## Supporting information


**Supporting Information**.

Supporting information.

Supporting information.

Supporting information.

## Data Availability

All relevant data are within the article, or within previously published articles, and supporting information files.
