# Supplementary material for: Acceptability of risk stratification within population‐based cancer screening from the perspective of the general public: A mixed‐methods systematic review
Source: Health Expect. 2023 Feb 28;26(3):989–1008. doi: 10.1111/hex.13739 (PMC10154794; doi:10.1111/hex.13739)
Supplement: Supplementary file 2 — Supporting information. [file HEX-26--s004.docx]

**Supplementary Table 2. Mixed Methods Appraisal Tool results for quality assessment.**

| Author (year): | Study type: | Relevant sampling strategy? | Representative sample? | Appropriate measurements? | Nonresponse bias? | Appropriate statistical analysis? | Appropriate approach? | Adequate methods? | Findings derived from data? | Substantiated interpretation? | Coherence? |
| --- | --- | --- | --- | --- | --- | --- | --- | --- | --- | --- | --- |
| Meisel (2015) (24) | Quantitative (descriptive) | 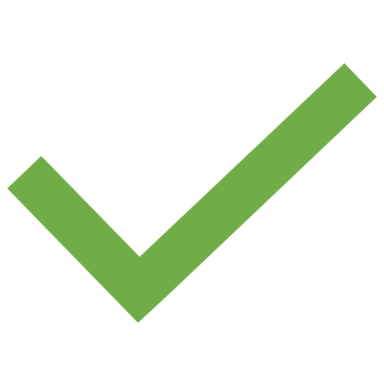 | 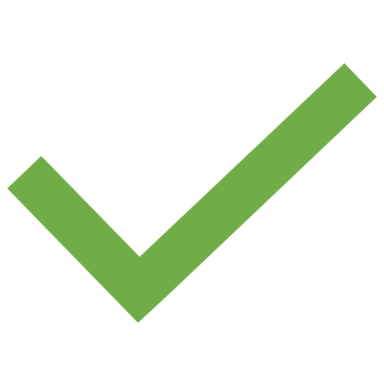 | 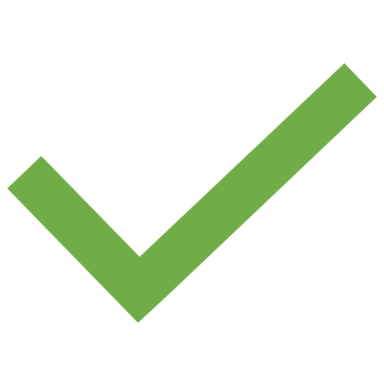 | 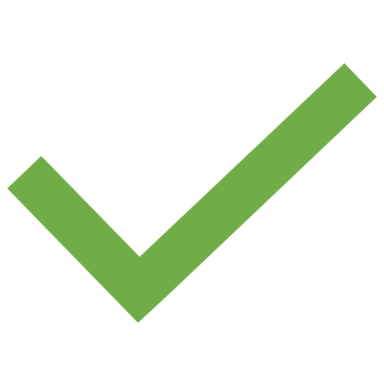 | 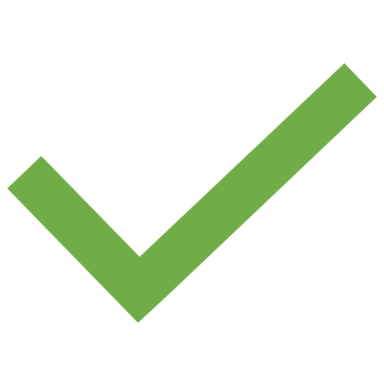 |  |  |  |  |  |
| Koitsalu (2016) (23) |  | 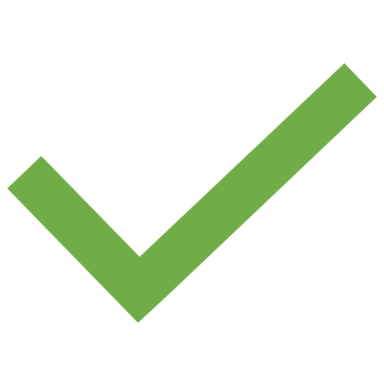 | 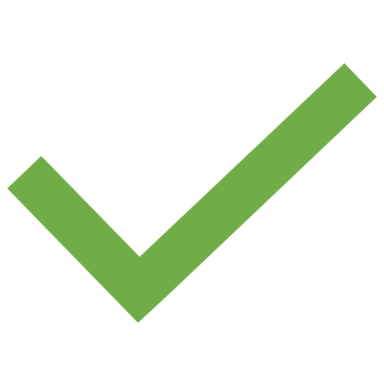 | 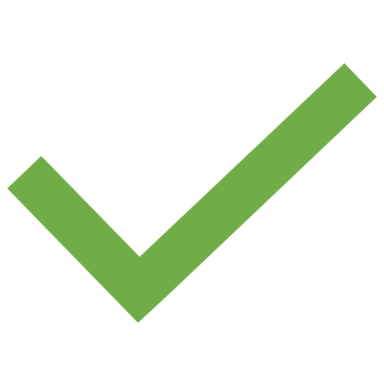 | 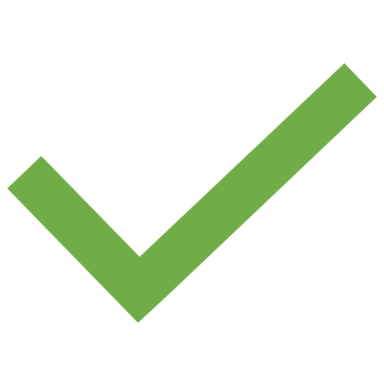 | 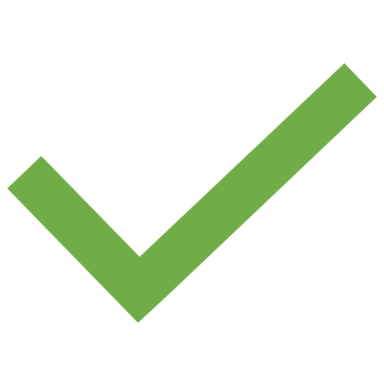 |  |  |  |  |  |
| Meisel (2016) (22) |  | 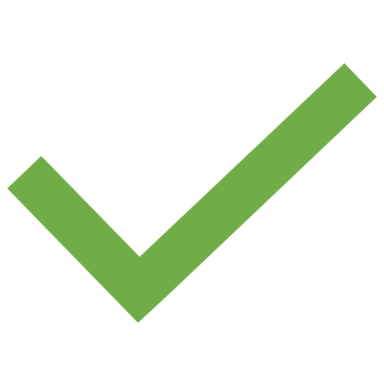 | 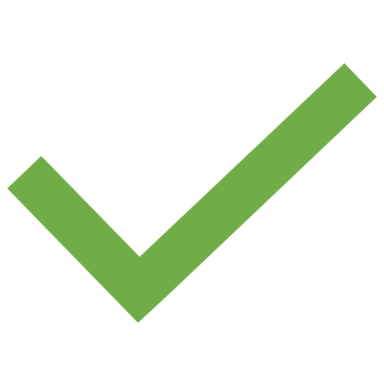 | 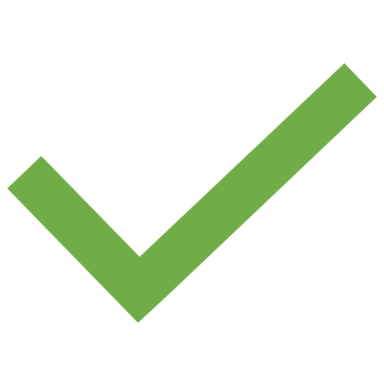 | 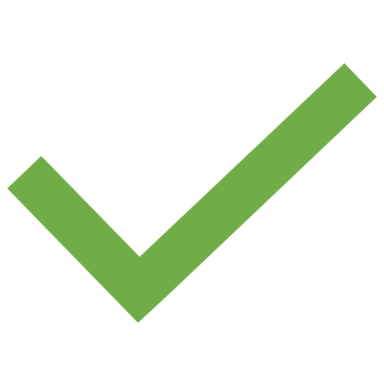 | 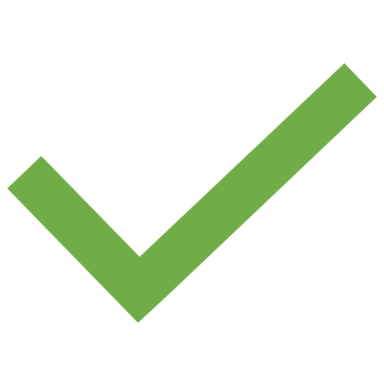 |  |  |  |  |  |
| Piper (2018) (26) |  | 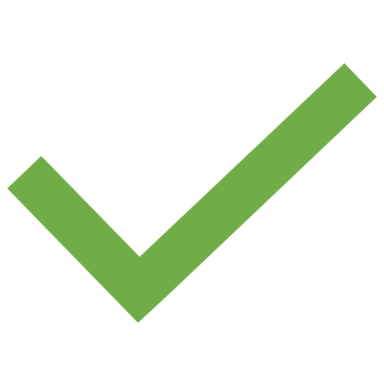 | 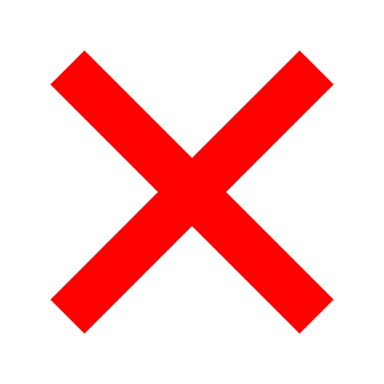 | 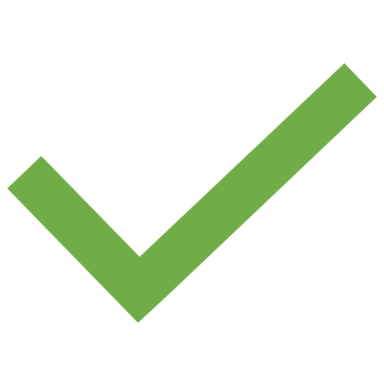 | 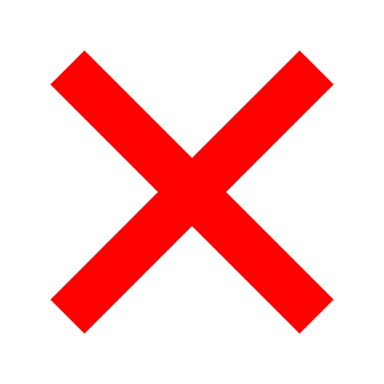 | 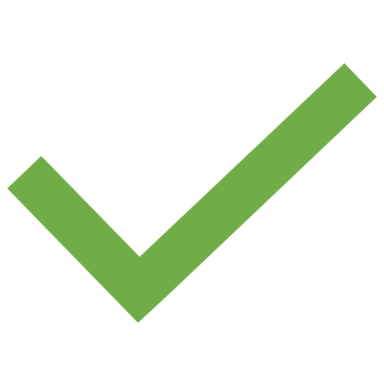 |  |  |  |  |  |
| Ghanouni (2020a) (5) |  | 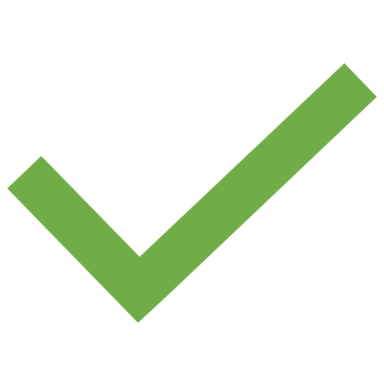 | 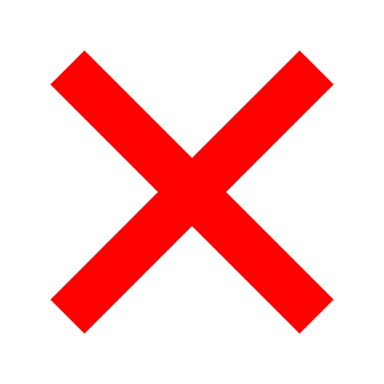 | 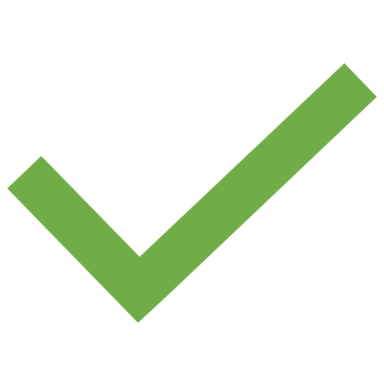 | 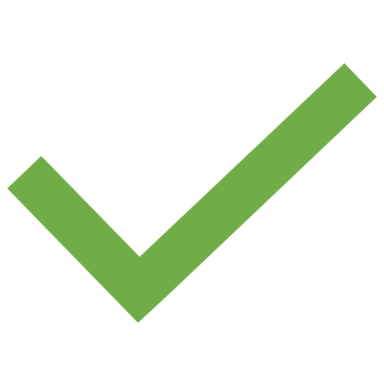 | 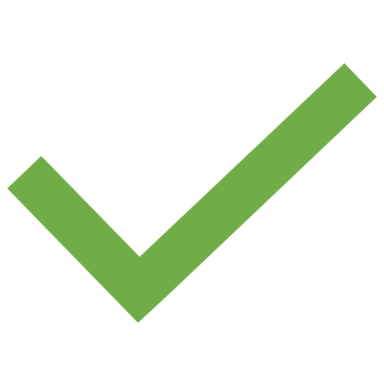 |  |  |  |  |  |
| Ghanouni (2020b) (25) |  | 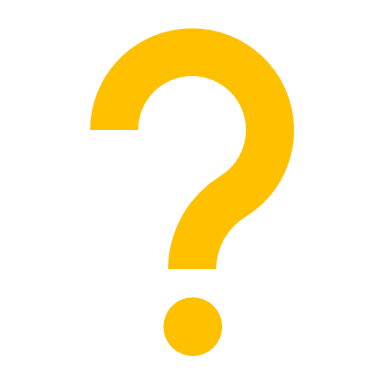 | 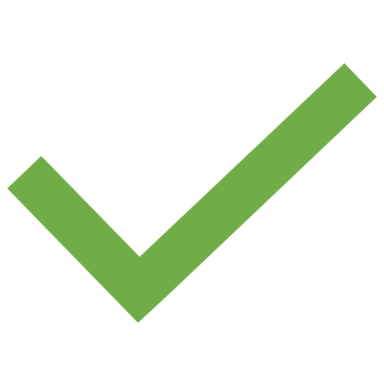 | 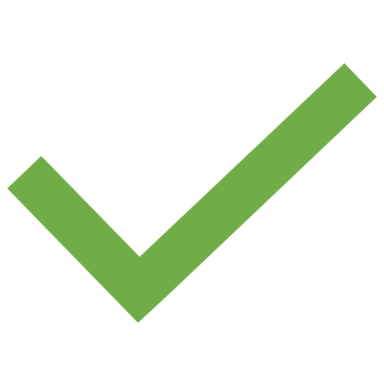 | 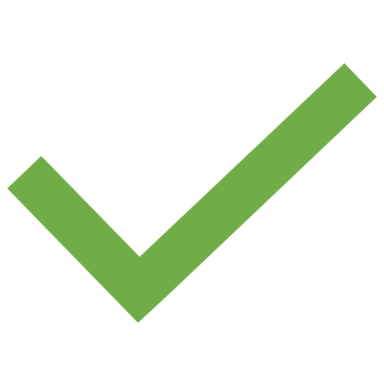 | 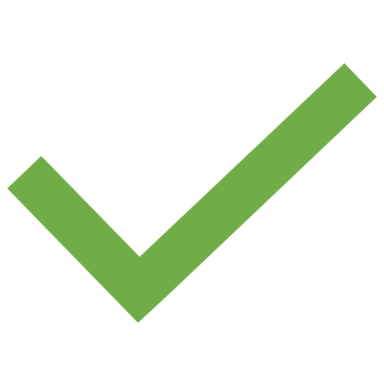 |  |  |  |  |  |
| Rainey (2020a) (21) |  | 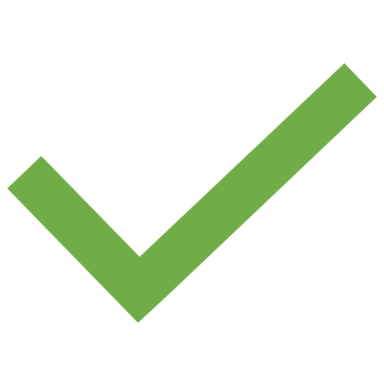 | 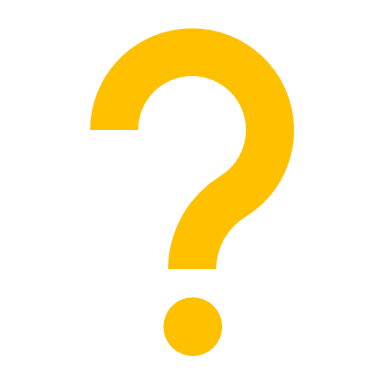 | 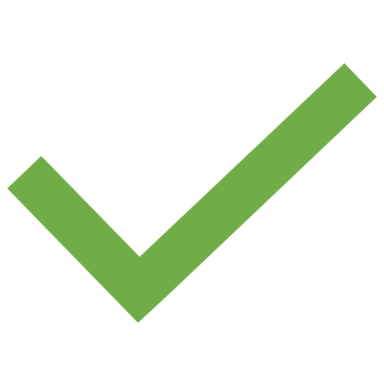 | 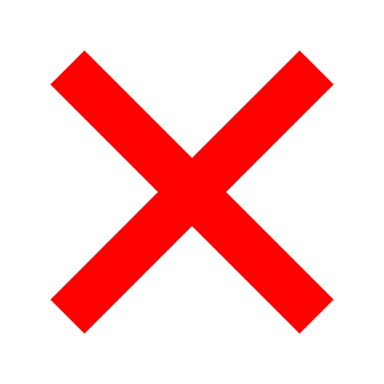 | 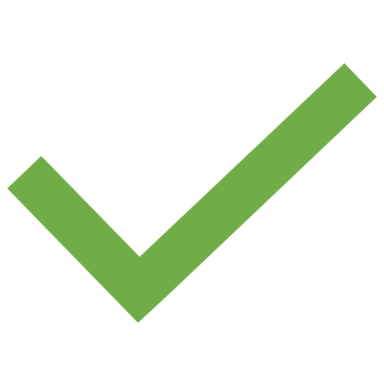 |  |  |  |  |  |
| Mbuya-Bienge (2021) (20) |  | 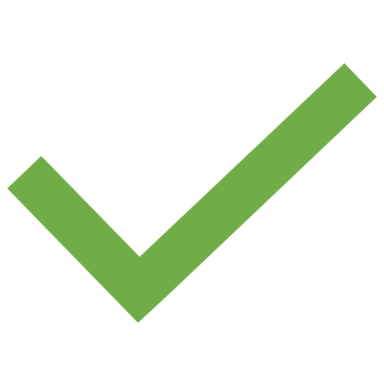 | 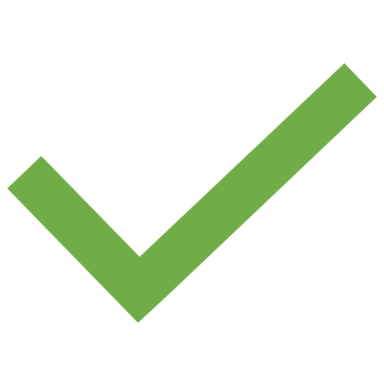 | 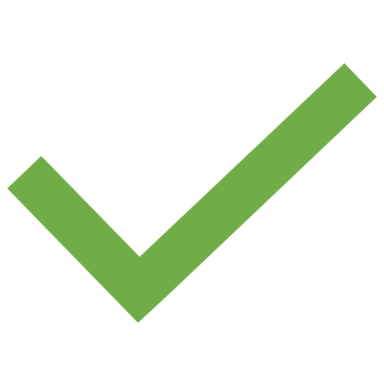 | 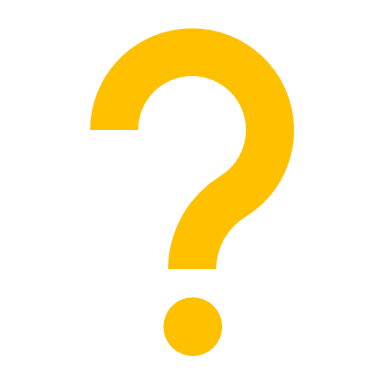 | 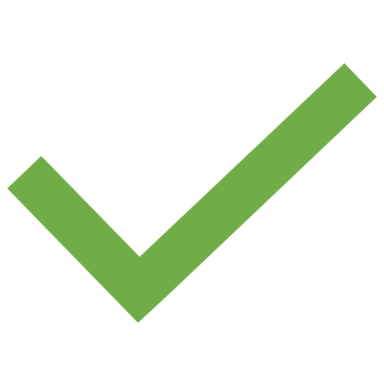 |  |  |  |  |  |
| Usher-Smith (2021) (19) |  | 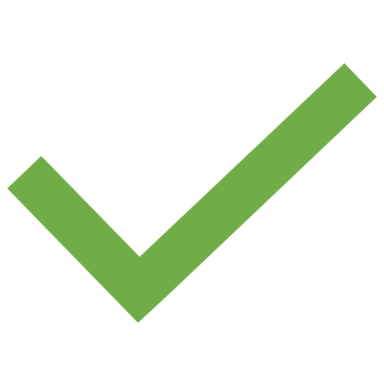 | 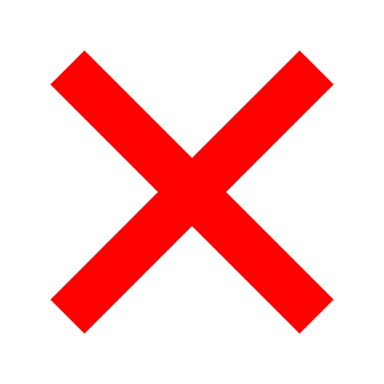 | 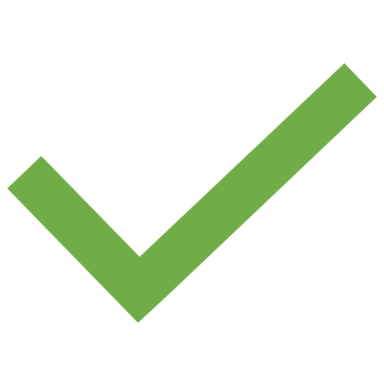 | 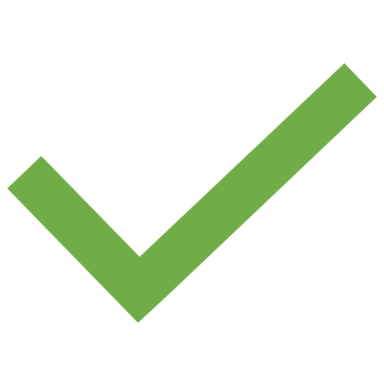 | 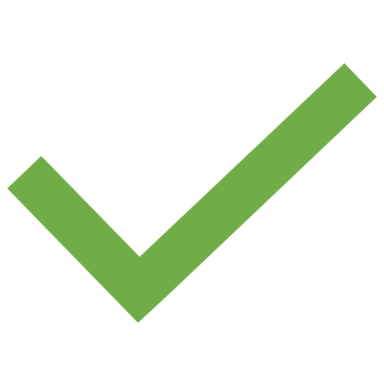 |  |  |  |  |  |
| Henneman (2011) (35) | Qualitative |  |  |  |  |  | 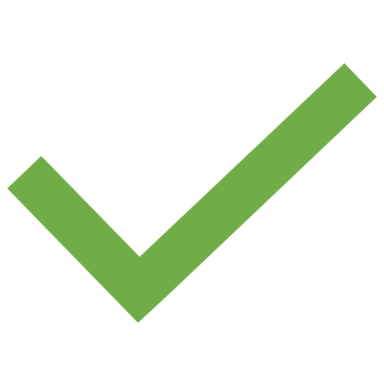 | 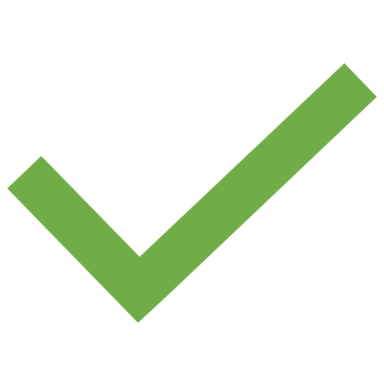 | 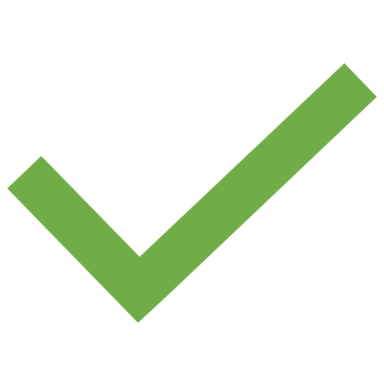 | 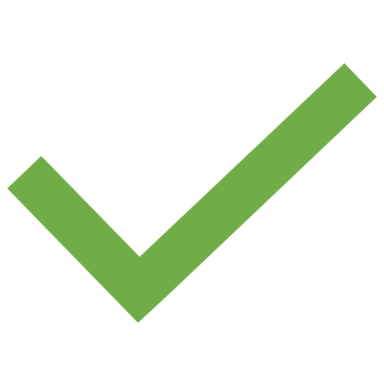 | 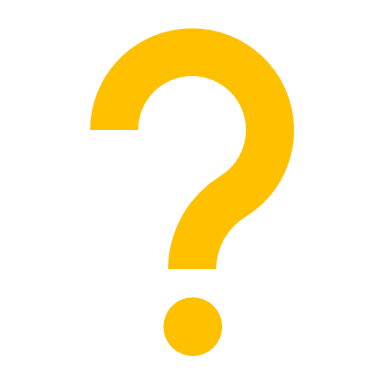 |
| Meisel (2013) (37) |  |  |  |  |  |  | 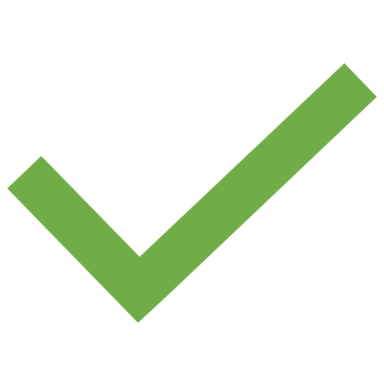 | 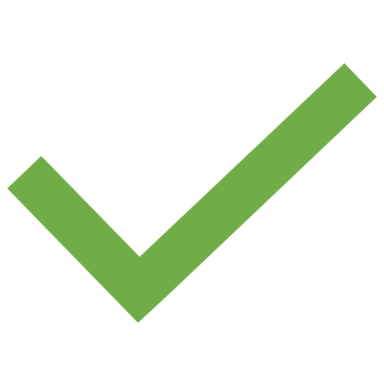 | 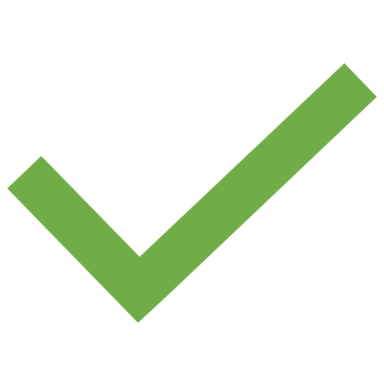 | 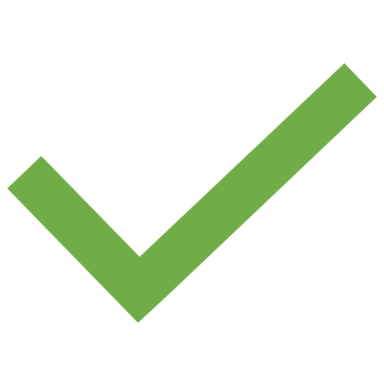 | 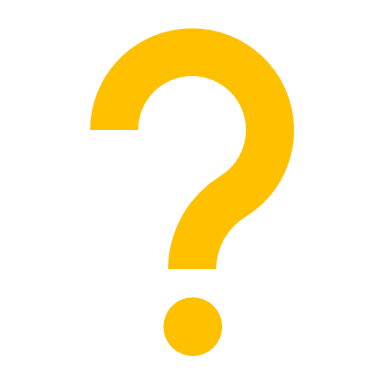 |
| Rahman (2015) (39) |  |  |  |  |  |  | 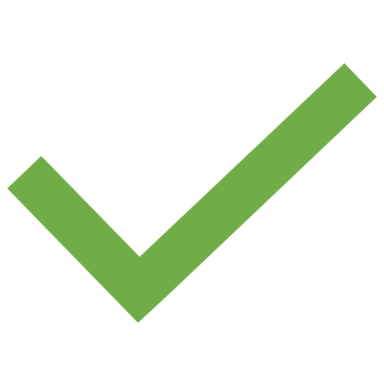 | 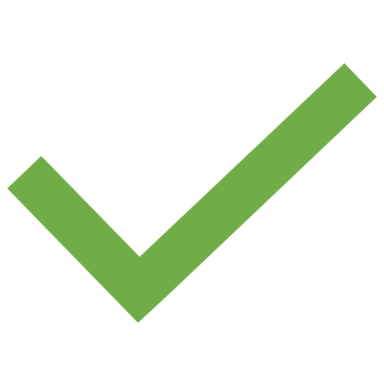 | 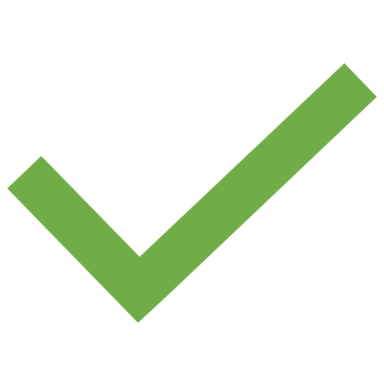 | 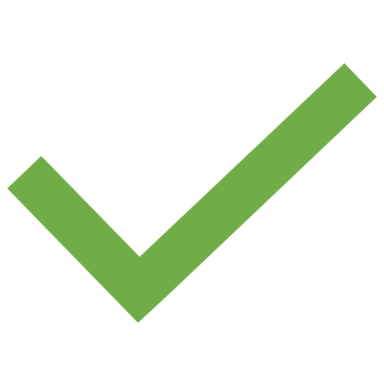 | 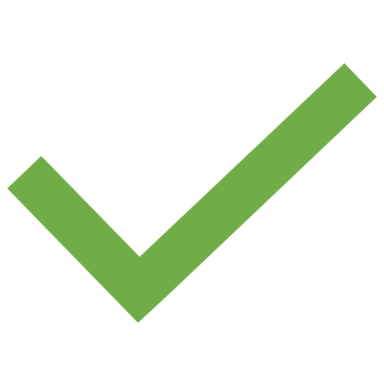 |
| Hann (2018) (33) |  |  |  |  |  |  | 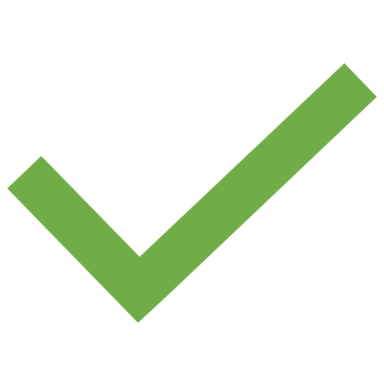 | 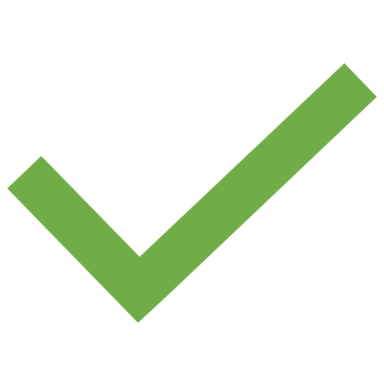 | 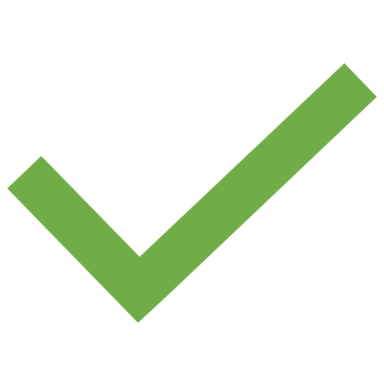 | 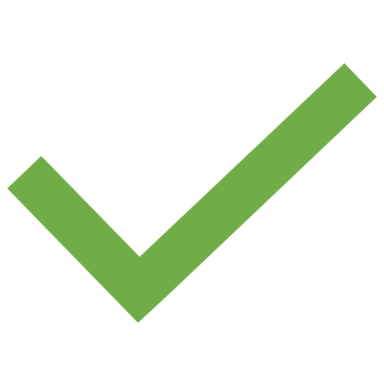 | 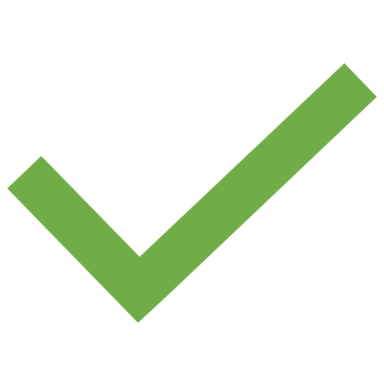 |
| He (2018) (34) |  |  |  |  |  |  | 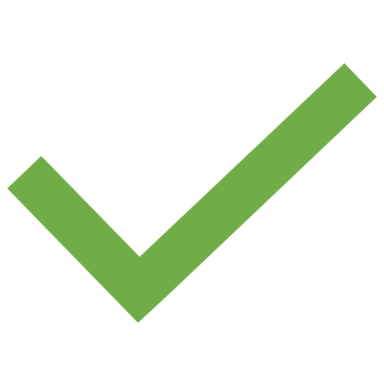 | 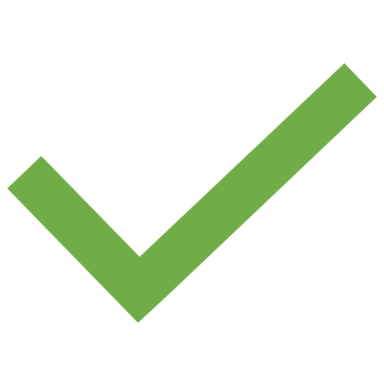 | 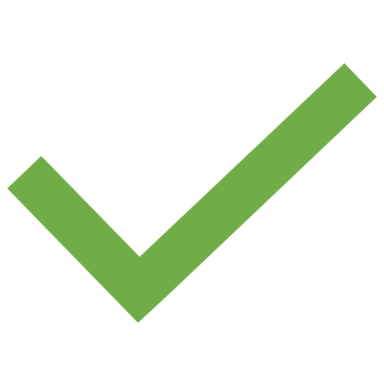 | 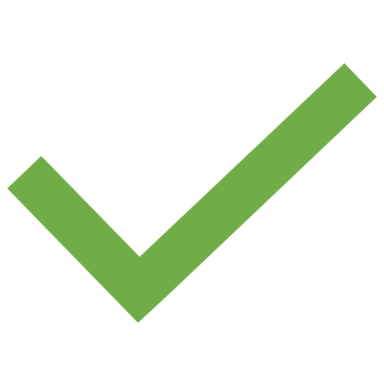 | 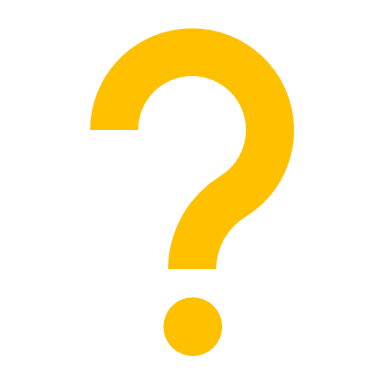 |
| Lippey (2019) (32) |  |  |  |  |  |  | 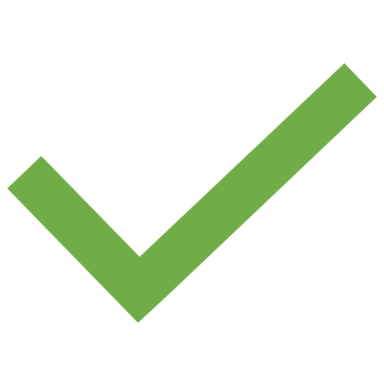 | 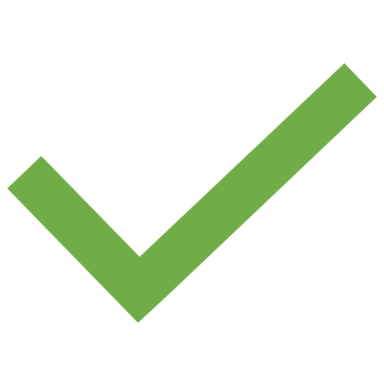 | 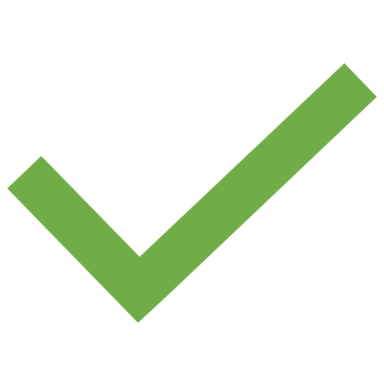 | 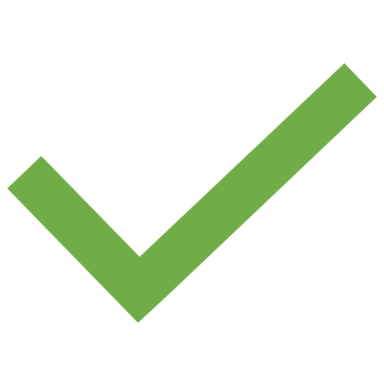 | 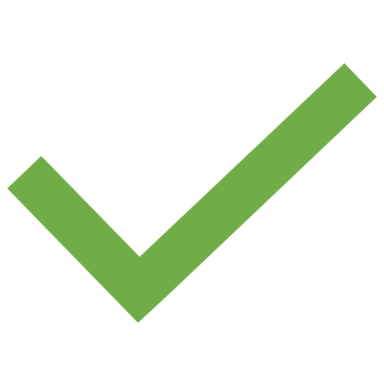 |
| Rainey (2019) (36) |  |  |  |  |  |  | 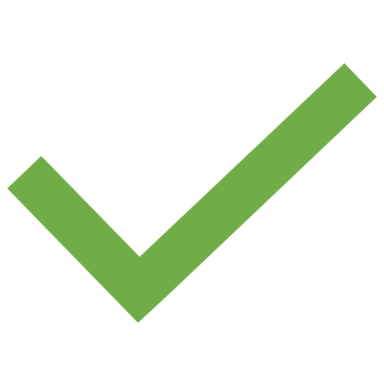 | 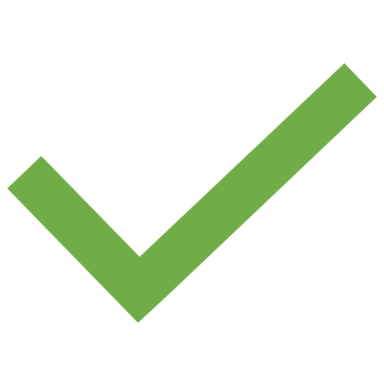 | 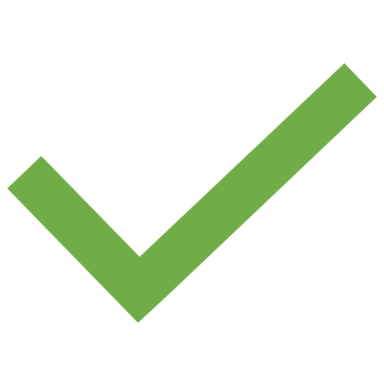 | 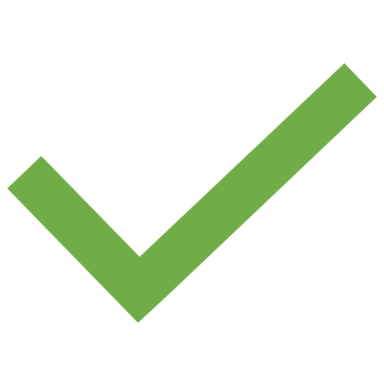 | 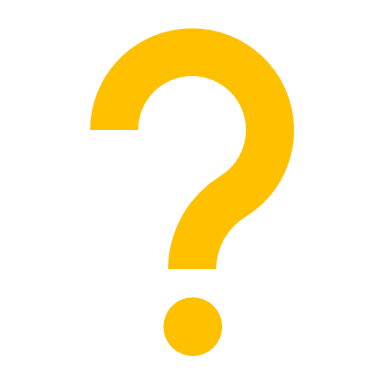 |
| Rainey (2020b) (31) |  |  |  |  |  |  | 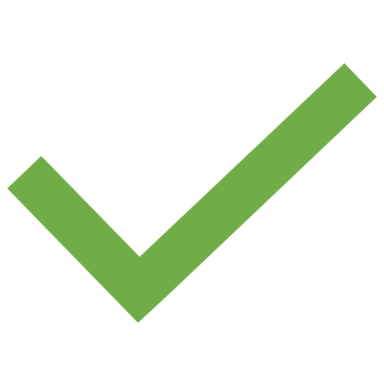 | 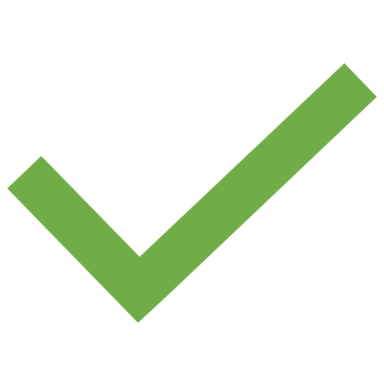 | 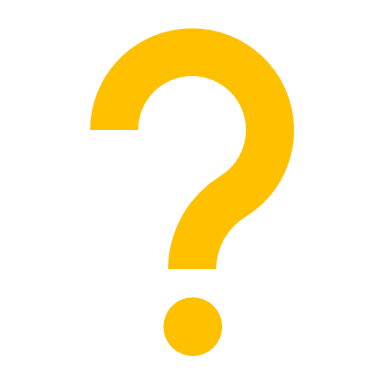 | 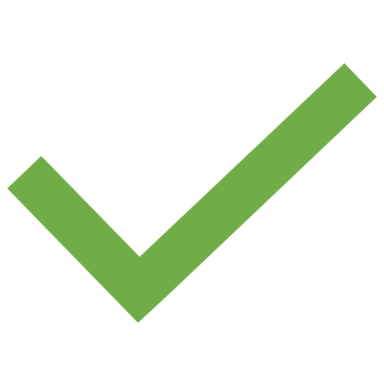 | 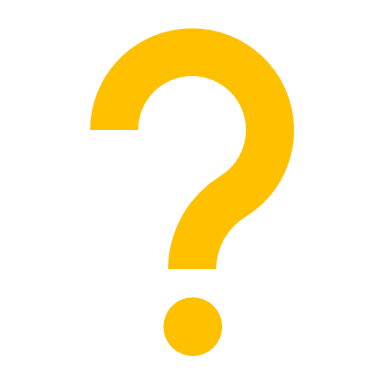 |
| Woof (2020) (28) |  |  |  |  |  |  | 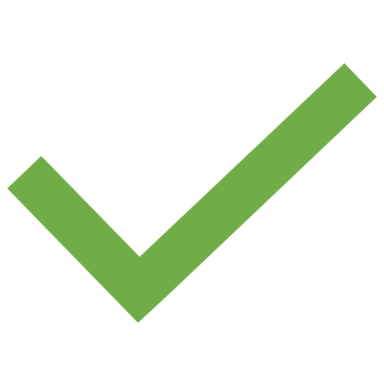 | 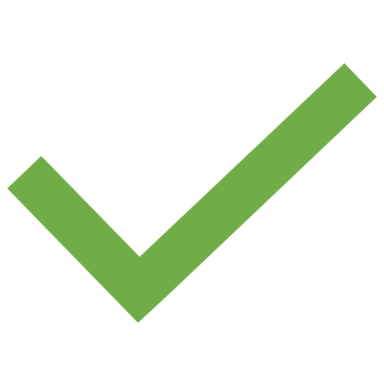 | 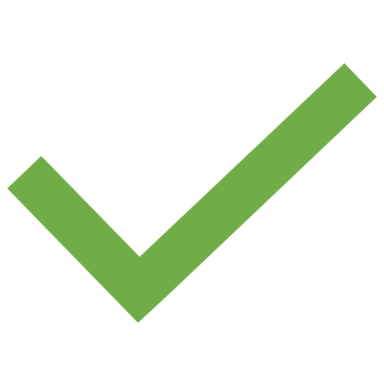 | 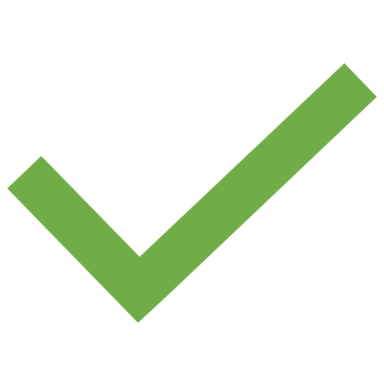 | 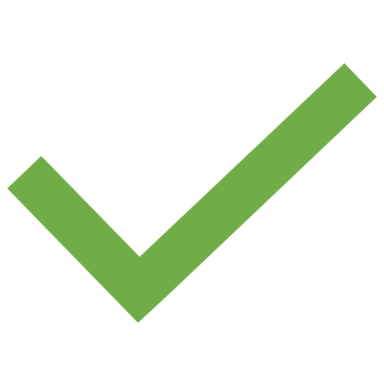 |
| Dunlop (2021) (30) |  |  |  |  |  |  | 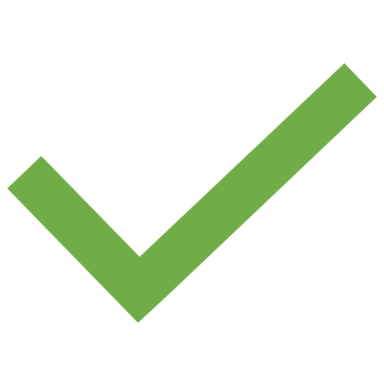 | 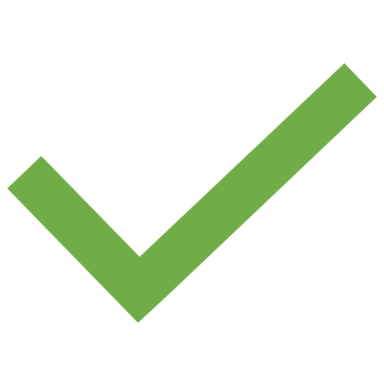 | 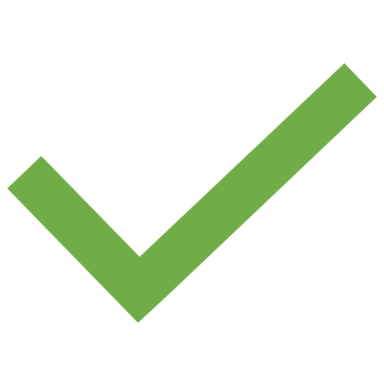 | 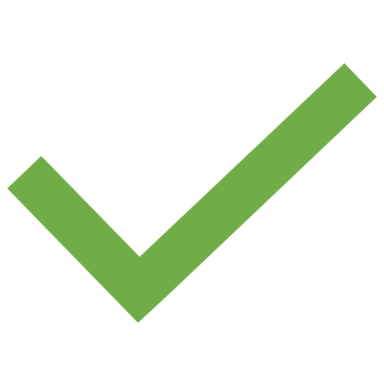 | 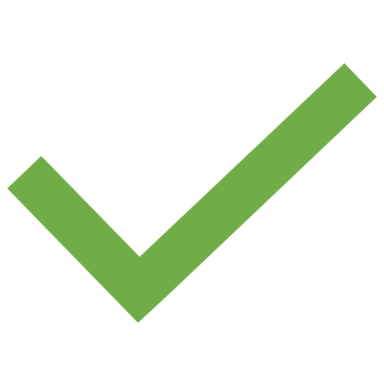 |
| Kelley-Jones (2021) (38) |  |  |  |  |  |  | 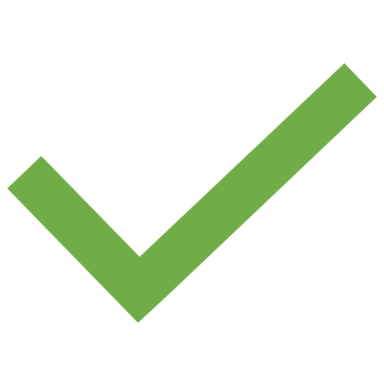 | 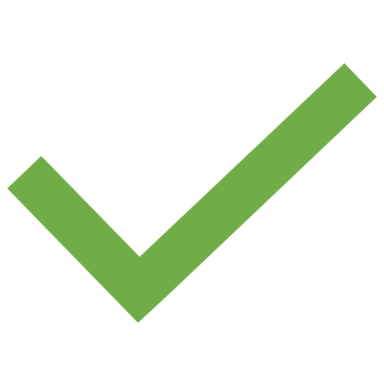 | 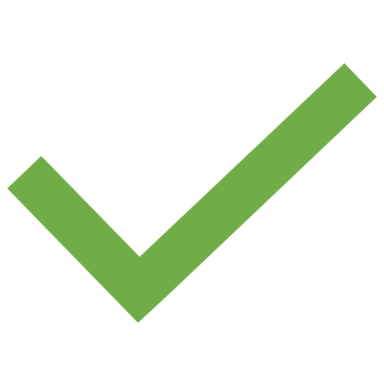 | 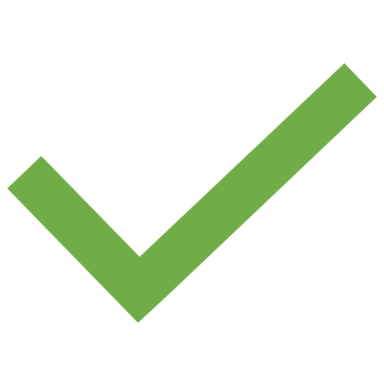 | 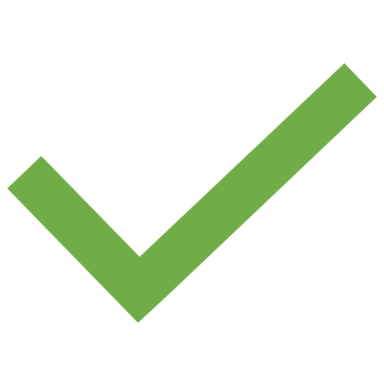 |
| McWilliams (2021) (27) |  |  |  |  |  |  |  |  |  |  |  |
| Sierra (2021) (29) |  |  |  |  |  |  |  |  |  |  |  |

*All papers scored ‘Yes’ for screening questions 1 & 2 (S1. Are there clear research questions? S2. Do the collected data allow to address the research questions?)*

*Yes*

*No*

*Can’t tell*
