# Supplementary material for: Acceptability of risk stratification within population‐based cancer screening from the perspective of the general public: A mixed‐methods systematic review
Source: Health Expect. 2023 Feb 28;26(3):989–1008. doi: 10.1111/hex.13739 (PMC10154794; doi:10.1111/hex.13739)
Supplement: Supplementary file 3 — Supporting information. [file HEX-26--s001.docx]

**Supplementary Table 3. Distribution of the evidence relating to each theme.**

|  | | Author (year): | | | | | | | | | | | | | | | | | | | | | | |
| --- | --- | --- | --- | --- | --- | --- | --- | --- | --- | --- | --- | --- | --- | --- | --- | --- | --- | --- | --- | --- | --- | --- | --- | --- |
|  |  | Meisel (2015) (24) | Koitsalu (2016) (23) | Meisel (2016) (22) | Piper (2018) (26) | Ghanouni (2020a) (5) | Ghanouni (2020b) (25) | Rainey (2020a) (21) | Mbuya-Bienge (2021) (20) | Usher-Smith (2021) (19) | Henneman (2011) (35) | Meisel (2013) (37) | Rahman (2015) (39) | Hann (2018) (33) | He (2018) (34) | Lippey (2019) (32) | Rainey (2019) (36) | Rainey (2020b) (31) | Woof (2020) (28) | Dunlop (2021) (30) | Kelley-Jones (2021) (38) | McWilliams (2021) (27) | Sierra (2021) (29) |  |
| MMAT result: | |  |  |  |  |  |  |  |  |  |  |  |  |  |  |  |  |  |  |  |  |  |  |  |
| Affective Attitude | General attitudes towards risk stratification |  |  |  |  |  |  |  |  |  |  |  |  |  |  |  |  |  |  |  |  |  |  |  |
|  | How to communicate risk estimates |  |  |  |  |  |  |  |  |  |  |  |  |  |  |  |  |  |  |  |  |  |  |  |
|  | The impact of knowing your risk |  |  |  |  |  |  |  |  |  |  |  |  |  |  |  |  |  |  |  |  |  |  |  |
| Burden | Barriers to accessibility |  |  |  |  |  |  |  |  |  |  |  |  |  |  |  |  |  |  |  |  |  |  |  |
|  | Emotional or psychological burden |  |  |  |  |  |  |  |  |  |  |  |  |  |  |  |  |  |  |  |  |  |  |  |
| Ethicality | The importance of prevention & early detection |  |  |  |  |  |  |  |  |  |  |  |  |  |  |  |  |  |  |  |  |  |  |  |
|  | Is risk stratification fair? |  |  |  |  |  |  |  |  |  |  |  |  |  |  |  |  |  |  |  |  |  |  |  |
|  | Cost as the motivation behind transitioning to risk stratification |  |  |  |  |  |  |  |  |  |  |  |  |  |  |  |  |  |  |  |  |  |  |  |
| Intervention Coherence | Risk stratification is logical in principle |  |  |  |  |  |  |  |  |  |  |  |  |  |  |  |  |  |  |  |  |  |  |  |
|  | Variation in understanding the evidence for risk stratification |  |  |  |  |  |  |  |  |  |  |  |  |  |  |  |  |  |  |  |  |  |  |  |
| Opportunity Cost | Cost of screening & of lifestyle changes |  |  |  |  |  |  |  |  |  |  |  |  |  |  |  |  |  |  |  |  |  |  |  |
|  | Data security, privacy, & the potential for discrimination |  |  |  |  |  |  |  |  |  |  |  |  |  |  |  |  |  |  |  |  |  |  |  |
| Perceived Effectiveness | Considerations for people at low risk |  |  |  |  |  |  |  |  |  |  |  |  |  |  |  |  |  |  |  |  |  |  |  |
|  | The impact of HCP involvement on implementation & delivery |  |  |  |  |  |  |  |  |  |  |  |  |  |  |  |  |  |  |  |  |  |  |  |
|  | The impact of risk assessment on wider outcomes |  |  |  |  |  |  |  |  |  |  |  |  |  |  |  |  |  |  |  |  |  |  |  |
| Self-efficacy | Feelings of personal responsibility |  |  |  |  |  |  |  |  |  |  |  |  |  |  |  |  |  |  |  |  |  |  |  |
|  | A need for help & guidance from HCPs |  |  |  |  |  |  |  |  |  |  |  |  |  |  |  |  |  |  |  |  |  |  |  |
|  | Willingness to participate in risk assessment |  |  |  |  |  |  |  |  |  |  |  |  |  |  |  |  |  |  |  |  |  |  |  |

*Large circle= ‘yes’ for all MMAT domains*

*Medium circle = ‘Can’t tell’ for one MMAT domain*

*Small circle = ‘no’ for one or more MMAT domains or ‘can’t tell’ for two or more MMAT domains*

*Clear = study briefly contributes to the theme*

*Filled = study strongly contributes to the theme*

HCP – healthcare professional

MMAT – Mixed Methods Appraisal Tool
